# Spatial transcriptomics in embryonic mouse diaphragm muscle reveals regional gradients and subdomains of developmental gene expression

**DOI:** 10.1016/j.isci.2024.110018

**Published:** 2024-05-17

**Authors:** Mehmet Mahsum Kaplan, Maximilian Zeidler, Annabella Knapp, Martina Hölzl, Michaela Kress, Helga Fritsch, Anne Krogsdam, Bernhard E. Flucher

**Affiliations:** 1 Institute of Physiology, Medical University Innsbruck, 6020 Innsbruck, Austria; 2 Institute of Clinical and Functional Anatomy, Medical University Innsbruck, 6020 Innsbruck, Austria; 3 Deep Sequencing Core and Institute for Bioinformatics Medical University Innsbruck, 6020 Innsbruck, Austria

**Keywords:** Molecular physiology, Developmental biology, Transcriptomics, Model organism

## Abstract

The murine embryonic diaphragm is a primary model for studying myogenesis and neuro-muscular synaptogenesis, both representing processes regulated by spatially organized genetic programs of myonuclei located in distinct myodomains. However, a spatial gene expression pattern of embryonic mouse diaphragm has not been reported. Here, we provide spatially resolved gene expression data for horizontally sectioned embryonic mouse diaphragms at embryonic days E14.5 and E18.5. These data reveal gene signatures for specific muscle regions with distinct maturity and fiber type composition, as well as for a central neuromuscular junction (NMJ) and a peripheral myotendinous junction (MTJ) compartment. Comparing spatial expression patterns of wild-type mice with those of transgenic mice lacking either the skeletal muscle calcium channel Ca_V_1.1 or β-catenin, reveals curtailed muscle development and dysregulated expression of genes potentially involved in NMJ formation. Altogether, these datasets provide a powerful resource for further studies of muscle development and NMJ formation in the mouse.

## Highlights

• Spatial atlas for gene expression in embryonic and fetal mouse diaphragm
• Documentation of novel gene expression spatially correlated with *Chrna1*
• Spatial alterations of NMJ genes in mice lacking Ca_V_1.1 or muscle β-catenin
• Spatial aspects of diaphragm muscle development controlled by β-catenin

## Graphical abstract

**Figure:**
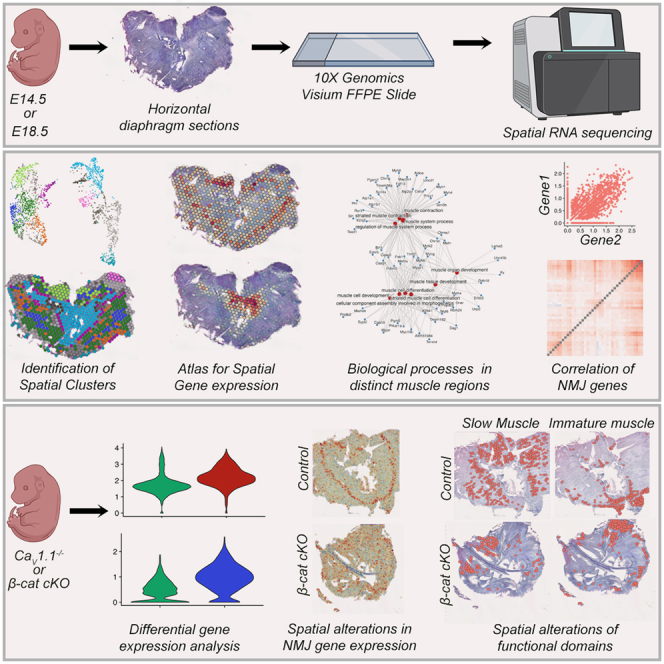

## Introduction

In multinucleated skeletal muscle fibers, nuclei are arrayed from end to end. Although sharing the same sarcoplasm, these nuclei have distinct transcriptional programs depending on their position in the muscle fiber. For example, nuclei close to the myotendinous junction (MTJ) express genes crucial for its function as a structural focal point connecting tendon and muscle.^1^ Likewise, nuclei underneath the neuromuscular junction (NMJ), which are located at the middle of the myofibers, express genes that are important for formation, function, and maintenance of nerve-muscle synapses.^2^^,^^3^^,^^4^ It is well established that spatial compartmentalization of myodomains during embryonic development is instrumental for the regulation of diverse and mutually interdependent developmental processes. For example, genes involved in myogenesis are vital also for neuromuscular synaptogenesis,^5^ while conversely synaptic transmission, muscle activity, and excitation-contraction coupling (ECC) initiated at the NMJ are critical for myogenesis^6^^,^^7^ and even for the proper development of the tendon,^8^ which in turn is required for correct muscle development.^9^ Therefore, it is important to spatially resolve the transcriptional signatures of functionally distinct compartments of developing muscle and tendon to better understand the regulation of these critical processes.

Taking advantage of the advances in single nucleus RNA-sequencing (snRNA-seq), single nucleus ATAC-sequencing (snATAC-seq) and spatial transcriptomics (ST), recent studies reported gene expression data for heterogeneous nuclei populations in adult muscle fibers and how particular types of nuclei transcriptionally responded to denervation, aging or muscular dystrophy.^10^^,^^11^^,^^12^^,^^13^^,^^14^^,^^15^^,^^16^ More recently, snRNA-seq and snATAC-seq of embryonic and adult hindlimb muscle revealed opposing genetic mechanisms for muscle development and maturation.^17^ While spatial transcriptomics in human embryonic limb^18^ and snRNA-seq in developing mouse diaphragms have been reported,^19^ a spatially resolved global gene expression profile of developing mouse diaphragm, which is widely used as a model for myogenesis and neuro-muscular development due to its accessibility, flat shape, and clear separation of anatomical structures mentioned previously, has hitherto not been reported. To this end, we studied spatial gene expression in mouse embryonic and fetal diaphragms. Our findings support distinct transcriptomic signatures of separable muscle domains at E14.5 and E18.5. Particularly, we identified centrally expressed genes which were highly correlated with AChR expression and thus could possibly be involved in NMJ formation. Furthermore, we demonstrated how the lack of critical regulators of muscle and NMJ formation, Ca_V_1.1 or β-catenin, impacts gene expression during late embryonic development in mouse diaphragm.

## Results

### Gene expression profiles of distinct anatomical structures in developing mouse diaphragms

#### Overview of the approach

**Figure 1:**
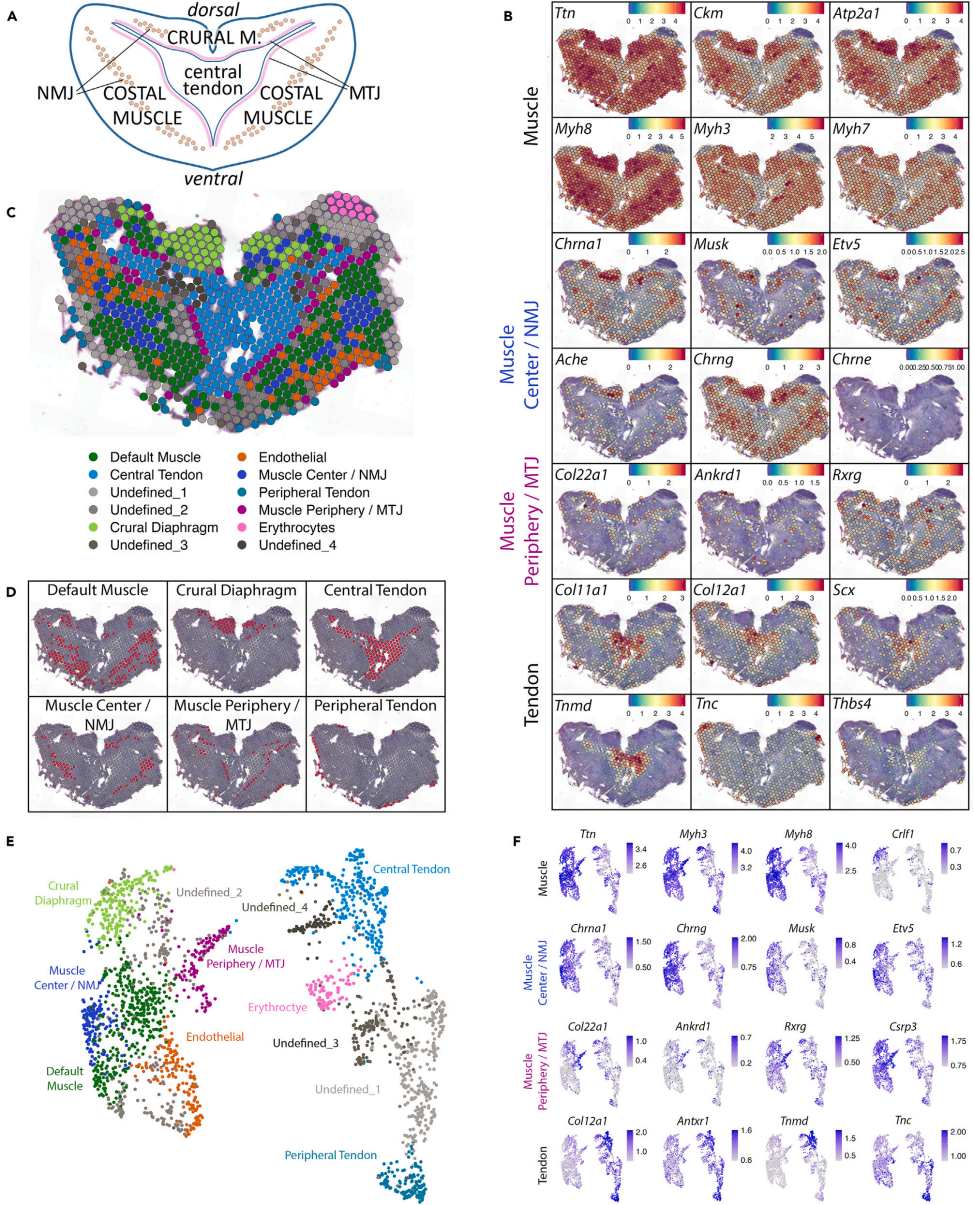
Spatial Transcriptomics in horizontally sectioned E14.5 mouse diaphragm identifies distinct tissues and muscle domains (A) Schematic representation of embryonic mouse diaphragm in which relevant anatomical regions are indicated. NMJ: Neuromuscular junction, MTJ: Myotendinous junction. (B) SpatialFeaturePlots demonstrating expression levels and distributions of representative genes for muscle, NMJ, MTJ and tendon. (C) SpatialDimPlot demonstrating the distribution of all the identified clusters within the diaphragm tissue. (D) Spatial distribution of representative Seurat clusters differentiating genetically specific domains in developing mouse diaphragm. (E) Uniform Manifold Approximation and Projection (UMAP) diagram of identified clusters of spatial RNA sequencing with muscle and endothelial clusters at the left and tendon and erythrocyte clusters at right. (F) FeaturePlots showing expression of muscle (*Ttn*, *Myh3*, *Myh8*), crural diaphragm (*Crlf1*), NMJ (*Chrna1*, *Chrng*, *Musk, Etv5*), MTJ (*Col22a1*, *Ankrd1*, *Rxrg*, *Csrp3*) and tendon (*Col12a1*, Antxr1, *Tnmd*, *Tnc*) markers displayed by UMAP.

To spatially analyze gene expression patterns in developing skeletal muscle, we utilized the *10X Genomics Visium FFPE* (formaldehyde-fixed paraffin-embedded) *spatial gene expression* platform on horizontal sections of E14.5 and E18.5 mouse diaphragms. Data presented for control diaphragms were integrated from three samples of E14.5 and two samples of E18.5 mouse embryos. One representative sample from each time point is shown to visualize spatial plots. Despite the spatial limitation of *10X Genomics Visium FFPE* which does not allow single cell resolution, 55 μm spot diameter and 100 μm between centers of spots were sufficient to identify spatially distinct anatomical regions with specific gene expression profiles within the embryonic diaphragm preparation. The number of Visium spots covered by the tissue sections, median reads, UMI counts, and their distribution across the tissues can be accessed in the Table S1 and in processed datasets (GSE244014). Figure 1A depicts the morphology of mouse diaphragm with the lateral costal muscles and the dorsal crural muscles flanking the central tendinous plate. The muscle fibers are radially oriented, spanning the entire length between the central tendon and the rib cage. The interface between the muscles and the central tendon, as well as peripherally toward the rib cage contains the myotendinous junctions. In their center, the muscle fibers contain a single neuro-muscular junction, resulting in an endplate band running in the middle of the muscles perpendicular to the orientation of the muscle fibers.

#### Assessment of markers with known distributions in E14.5 diaphragm

To assess the validity of our method we first visualized genes with known distributions within the E14.5 diaphragm. Expression of known muscle genes (*Ttn*, *Ckm*, *Atp2a1*) and the embryonic myosin heavy chain isoforms (*Myh8*, *Myh3*) characterized the entire muscle domain, including costal and crural muscles (Figure 1B); *Myh3* and *Myh7* were expressed higher at the muscle periphery (Figure 1B) as previously published for day E15.5 forelimb muscle fibers.^10^ Also, a number of genes involved in NMJ formation and function (*Chrna1*,^20^ *Musk*^21^, *Etv5*,^22^ *Ache*^23^, *Chrng*^24^) (Figure 1B) were enriched near the muscle center. Consistent with the developmental stage, the epsilon subunit of mature nicotinic acetylcholine receptors (AChR; *Chrne*) (Figure 1B) was not detected.^24^ MTJ-associated genes *Col22a1*^25^ and *Ankrd1*^26^ (Figure 1B) were expressed at the perimeters of the muscle domains. Consistent with the location of the central tendon the middle of the diaphragm was dominated by tendon genes (*Col11a1*,^27^ *Col12a1*,^28^ *Scx*,^29^ *Tnmd*^30^) (Figure 1B). Overall, these spatial expression patterns recapitulated the known morphology and developmentally expressed genes of mouse diaphragm and demonstrate an accurate and sound methodology.

#### Identification of the spatially distinguished clusters in E14.5 diaphragm

We performed integration, dimensionality reduction, clustering, and visualization for three E14.5 diaphragms (Figure S1A) using the standard workflow of *Seurat*^31^, and used the *Clustree R*^32^ package (0.5.0) to determine the optimal cluster number according to developer’s instructions. This approach revealed 12 spatially resolved clusters with distinct expression patterns (Figures 1C–1E). Based on their location in the diaphragm and the expression of known marker genes, eight clusters could be unequivocally identified (Figures 1 and S1B). Four of these were classified as belonging to muscle tissue. First, “Crural Diaphragm” displayed the characteristic localization in the dorso-medial sector of the tissue and expressed *Crlf1* as top marker gene. *Crlf1* mRNA expression was specific for the crural diaphragm and absent from costal diaphragm (Figures 1F, S1B and S1C). Second, “Muscle Center/NMJ” was characterized by NMJ-related genes and was located near the center of the costal diaphragm (Figures 1C–1F and S1B). Extracting markers for this cluster resulted in 347 highly expressed genes in the central muscle domain where normally NMJ formation is initiated (Table S2). These included well known NMJ genes and genes recently identified to be associated with the NMJ by snRNA-seq studies in adult muscles (such as *Prkar1a*, *Ufsp1*, *Apobec2*)^10^^,^^11^^,^^12^ as well as congenital myasthenic syndrome associated NMJ gene *Scn4a*.^33^^,^^34^ In addition, novel genes were identified, which were hitherto not known to be expressed in this domain, such as another voltage-gated sodium channel *Scn3b***,** two mTOR signaling inhibitors *Ddit4l*^35^ and *Prkaa2*^36^ and *Tead4* which interacts with myogenin during myogenesis^17^ (Figure S1C; Table S2). Third, “Muscle Periphery/MTJ” was marked by 186 genes including known MTJ genes such as *Col22a1* and *Ankrd1*. Fourth, “Default Muscle” cluster was localized within the costal diaphragm, on both sides between central and peripheral cluster. This cluster expressed *Myh8*, *Ckm*, and *Atp2a1* as top markers (Figure S1B) and displayed a default muscle rather than a specialized transcriptomic signature like the other muscle clusters (Figure S2).

In addition to the muscle regions, we also could annotate two tendon clusters. “Central tendon” was located in the middle of the diaphragm, whereas “Peripheral Tendon” was laterally encircling the diaphragm (Figures 1C and 1D). Both of these clusters expressed known tendon genes (like *Col12a1*, *Antxr1*); yet they also displayed differential gene expression (DEG) profiles (Figure S1B; Table S2), documenting different genetic programs for central and peripheral parts of the diaphragmatic tendon. For example, *Tnmd* expression was specific to central tendon, whereas *Tnc*^37^ and *Thbs4* expression was more specific to peripheral tendon (Figures 1B–1F and S1B). In addition, clusters expressing known endothelial cell markers (such as *Pecam1*, *Kdr*, *Cdh5*) and erythrocyte markers (such as hemoglobins) were identified. The endothelial cluster also expressed lower levels of skeletal muscle markers (Figure S1B), a mixture of tissues, which is to be expected from the blood vessels distributed throughout the muscle tissue. Altogether, these data characterize spatially separable transcriptomic signatures of various cell types in horizontally sectioned embryonic mouse diaphragms and within the muscular portion reveals three functionally distinct subregions along the muscle fibers at E14.5. The expression patterns of the latter are indicative of their dual roles in muscle function and attachment to the tendon or formation of NMJs, respectively.

### Spatially divergent muscle differentiation in E14.5 diaphragms

#### Expression gradients of developmental markers indicate a center-to-periphery progression of myogenesis

**Figure 2:**
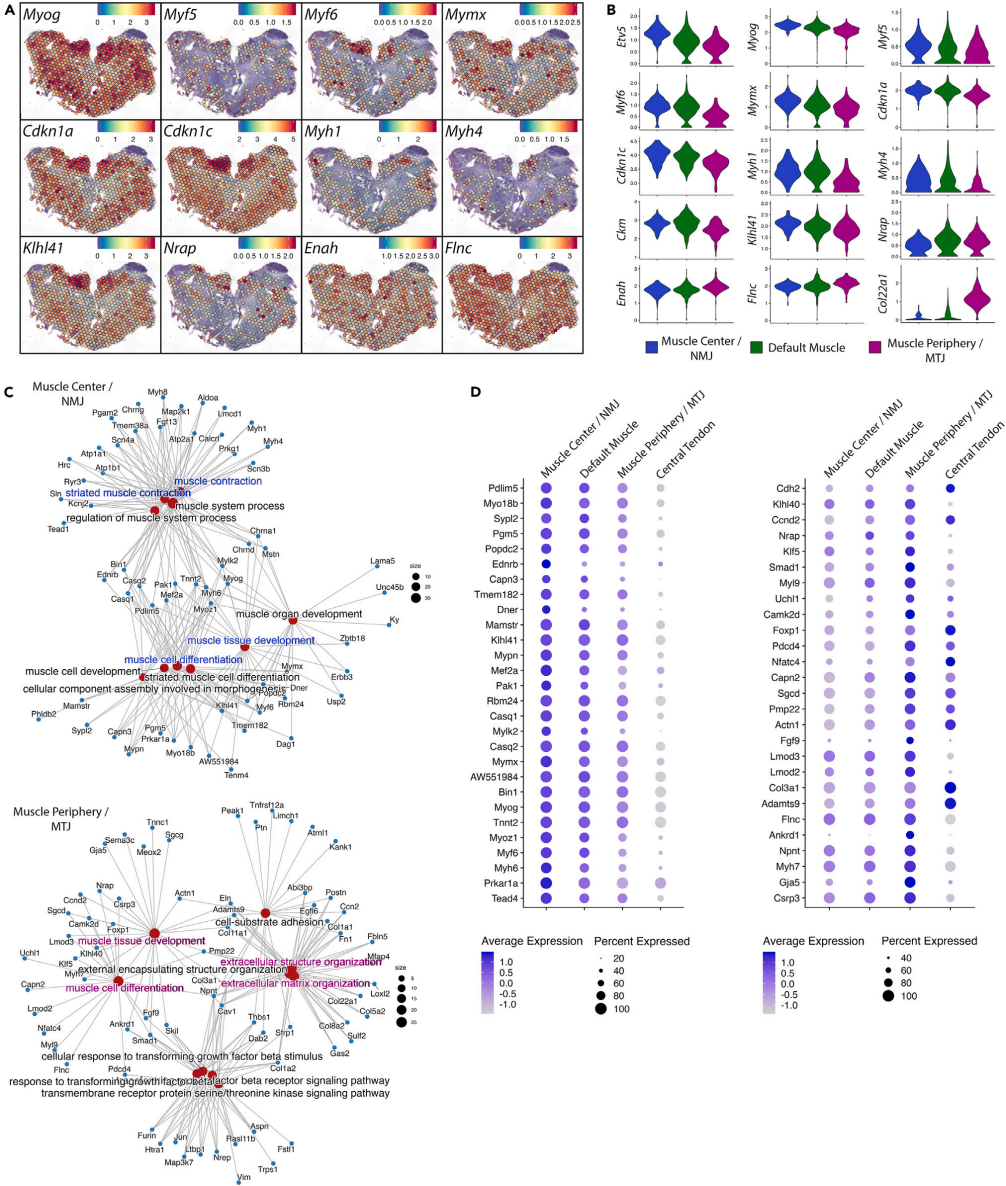
Spatial Transcriptomics reveals distinct myogenic processes in the muscle center and periphery in E14.5 mouse diaphragm (A) SpatialFeaturePlots demonstrating expression levels and distributions of myogenic differentiation markers. (B) VlnPlots of expression levels of genes in clusters identified as muscle center, default muscle, and muscle periphery show an overall increase or decrease in expression of genes involved in muscle development from the center to the periphery. Y axis indicates expression levels. (C) Cnetplots showing GO terms in biological processes (red nodes) and their associated genes (blue nodes) for the upregulated genes in clusters annotated as muscle center compared to muscle periphery (top) or muscle periphery compared to muscle center (bottom). (D) Dotplots of expression of genes involved in myogenesis displaying a declining (right) or increasing (left) gradient in clusters from the muscle center, over default muscle (muscle middle), to the muscle periphery.

Expression of a number of essential transcripts for terminal differentiation of muscle (*Myog*, *Myf5*, and *Myf6*),^38^ myoblast fusion (*Mymx*),^39^ cell cycle withdrawal during myogenesis (*Cdkn1a*^40^^,^^41^ and *Cdkn1c*^41^), end-state myonuclear differentiation factors (*Myh1*, *Myh4*, and *Ckm*)^12^ was lower at the peripheral MTJ cluster compared to the central NMJ cluster (Figures 2A and 2B), suggesting a differential progression of muscle differentiation between ends and the center of the fibers. Expression of the immature muscle fiber marker *Myh3*^42^ (Figure 1B) and genes associated with early myofibrillogenesis (*Nrap*, *Enah*, *Flnc*)^12^^,^^43^ was higher in the periphery, i.e., the MTJ. *Klhl41*, a crucial factor for degradation of Nrap during myofibril maturation,^44^ marked the central cluster. Fast muscle fiber type markers *Myh1* and *Myh4*, which replace *Myh3* and *Myh7* during muscle type specification, were detectable in the center of the muscle and this indicates that muscle fiber specification is already ongoing as early as E14.5. Most of the expressed genes showed gradients increasing from muscle center to periphery or vice versa with intermediate expression levels in the default muscle cluster in between (Figures 2A and 2B). Together, our data clearly indicated a progression of muscle fiber differentiation from the central region towards the muscle periphery with fiber type specification commencing centrally at E14.5. Numerous differentiation markers were highly expressed in the crural muscle cluster (Figure 2A), indicating an even more advanced differentiation of crural muscles compared to costal muscles.

#### Regulation of myogenesis by distinct factors in muscle center and periphery

To describe the biological processes taking place specifically in the muscle center and periphery, we performed DEG analysis between central and peripheral clusters followed by gene ontology (GO) enrichment analysis in biological processes terms for DEGs using the *clusterProfiler R* package (4.6.0).^45^ GO for genes upregulated in the central cluster revealed terms related to muscle contraction (GO:0006936) as well as neuromuscular processes (GO: 0050905) and development (GO:0007528). GO for genes upregulated in the peripheral cluster revealed the terms enriched extracellular matrix structure and organization (GO:0030198, GO:0043062) (Figure 2C; Table S3). These data highlight genes involved in the assembly of NMJ and MTJ structures captured by spots at the center and periphery, respectively. In addition to identified GO terms, GO analysis using DEGs between central and peripheral clusters also resulted in shared terms in biological processes, related to muscle cell differentiation (GO:0042692) and development (GO:0055001). However, in the two clusters these terms were brought about by different sets of genes involved in myogenesis, suggesting that distinct myogenic programs are active in the center and periphery of the muscle at the time of tissue preparation (Figure 2C). Interestingly, genes associated with muscle cell differentiation and development GO terms (Table S3) for the center (Figure 2D, left) and the periphery (Figure 2D, right) displayed a gradient increasing or decreasing between these two muscle regions. Moreover, *Taed4* and *Klf5*, which were recently identified by snRNA-seq to form a transcriptional complex with myogenin during embryonic development,^17^ also displayed spatial segregation in that *Tead4* expression was enriched in the muscle center whereas *Klf5* in the periphery (Figure 2D). In fact, we noted that spatial segregation of *Tead4* and *Klf5* was observable also in the snRNA-seq E14.5 data.^17^ Because gene expression data above suggest a more advanced differentiation in the muscle center, the distinct expression of genes involved in myogenesis likely represent their functions at different stages of muscle development. However, it must be noted that, due to large capture spot size (55 μm), in transition areas between muscle and tendon, muscle mRNAs are expected to be diluted by the mRNAs from tenocytes which can result in myogenic genes to be underrepresented in peripheral MTJ cluster. Nevertheless, intermediate expression levels of many of the critical myogenic genes in the default muscle cluster located between NMJ and MTJ (Figure 2B) and detection of other myogenic genes highly expressed in the peripheral domain compared to the rest of the muscle (Figure 2D, right) strongly suggest that myogenic processes proceed from central to peripheral muscle where they are controlled by distinct sets of genes. Moreover, the majority of myogenic genes showing increasing gradients from center to periphery do not correlate with high expression in the central tendon, excluding the possibility that their gradient is due to a contamination by tenocyte mRNAs (Figure 2D, right). This further indicates their involvement in myogenesis through their specific enrichment in the muscle periphery.

### Spatially divergent muscle differentiation in E18.5 diaphragms

#### Assessment of markers with known distributions

**Figure 3:**
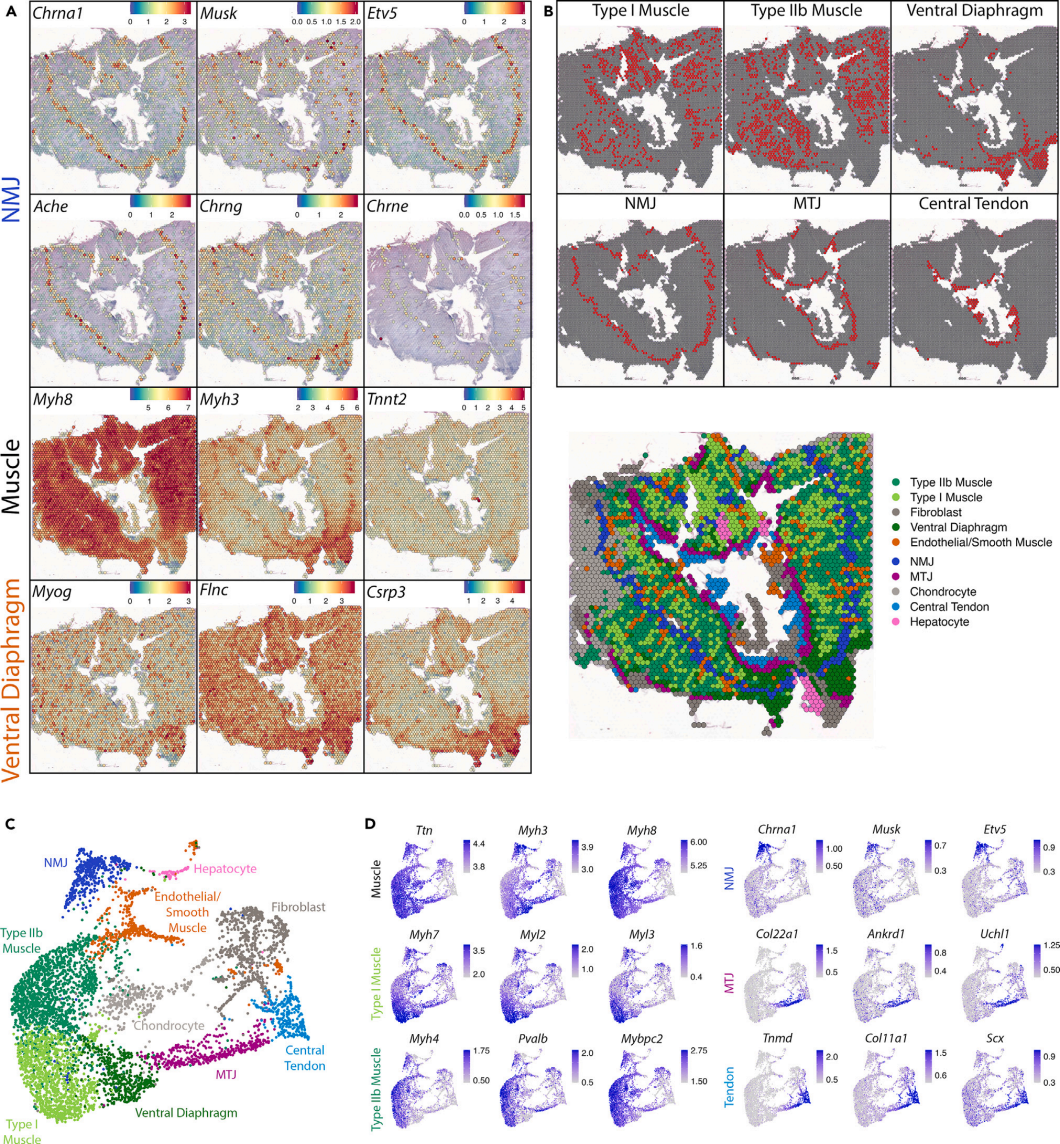
Spatial Transcriptomics in horizontally sectioned E18.5 mouse diaphragm identifies specific functional muscle domains and fiber types (A) SpatialFeaturePlots demonstrating expression levels and distributions of NMJ genes (*Chrna1*, *Musk*, *Etv5*, *Ache*, Chrng, *Chrne*), neonatal (*Myh8*) and embryonic (*Myh3*) myosin heavy chains, developmental troponin *Tnnt2*, and ventral diaphragm markers (*Myog*, *Flnc*, *Csrp3*). (B) Spatial distribution of Seurat clusters of distinct muscle and tendon domains. (C) UMAP representation of identified clusters of spatial RNA sequencing indicates spatially and functionally divergent differentiation of diaphragm muscle. (D) FeaturePlots demonstrating expression of muscle (*Ttn*, *Myh3*, *Myh8*), type I muscle (*Myh7, Myl2, Myl3*), type IIb muscle (*Myh4*, *Pvalb*, *Mybpc2*), NMJ (*Chrna1*, *Musk*, *Etv5*), MTJ (*Col22a1*, *Ankrd1*, *Uchl1*) and tendon (*Tnmd*, *Col11a1*, *Scx*) markers displayed by UMAP.

To determine the progression of gene expression during late embryonic to fetal development of mouse diaphragm, we performed spatial transcriptomics and analyses using two E18.5 diaphragm samples (Figure S3A) and compared the results with those of E14.5 diaphragms. The transcript expression pattern was similar to the known tissue distribution as expected at this developmental stage.^20^^,^^21^^,^^22^^,^^23^^,^^24^^,^^25^^,^^26^^,^^29^^,^^30^ For example, NMJ gene transcripts were specifically expressed in a narrow endplate band located at the center of the radially oriented muscle fibers and included low levels of the mature acetylcholine receptor isoform *Chrne*^24^ (Figure 3A). Interestingly, while the fetal/neonatal *Myh8* isoform was uniformly expressed throughout the muscle clusters, the embryonic *Myh3* was upregulated in both the NMJ and MTJ region (Figure 3A). This is in line with previous experimental data where *Myh3* transcripts were detected at both the muscle center and periphery of P2 forelimb muscles.^10^ Such distribution of *Myh3* is proposed to be transient in newly accrued myonuclei during fetal (secondary) myotube growth in these regions.^10^ The developmental troponin T isoform (*Tnnt2*) displayed a similar pattern (Figure 3A). *Myh3* distribution in the muscle center was remarkably correlated with that of *Chrna1* (Figure 3A) whose expression is under control of motor innervation at this developmental stage,^2^ which can be explained by the demonstrated association between sites of innervation and secondary myotube formation in murine muscle.^46^ Therefore, we suppose that there is a common mechanism for NMJ development and secondary myotube formation regulated upon motor innervation.

#### Identification of the spatially distinguished clusters in E18.5 diaphragm

The E18.5 mouse diaphragms revealed 10 transcriptomic clusters (Figures 3B, 3C, and S3B), which partially overlapped with those identified at E14.5. Clusters of non-muscle and non-tendon tissues included “Fibroblast” (*Clec3b*, *Sulf1*), “Hepatocyte” (*Alb*, *Ashg*, *Apoa1*), “Chondrocyte” (*Col2a1*, *Col9a1*, *Col9a3*) and one cluster which expressed a mixed signature of endothelial (*Ptprb*, *Cdh5*, *Pecam1*) and smooth muscle cells (*Tagln*, *Acta2*, *Myh11*) (Table S4). This mixed gene expression signature at E18.5, compared to the exclusively endothelial cluster at E14.5, supports a sequential recruitment of first endothelial and then smooth muscle cell during muscle vascularization.^47^

At E18.5, we again found specialized muscle and tendon clusters (“NMJ”, “MTJ”, and “Central Tendon”) similar to E14.5 diaphragms. Interestingly, crural diaphragm was no longer discerned as a distinct cluster at E18.5 (Figure 3B), yet *Crlf1* expression was still specific to crural diaphragm (Figure S3C). In addition to NMJ and MTJ, the muscle domain in E18.5 diaphragm was separated into three further distinct clusters. Two of these clusters appeared scattered between NMJ and MTJ (Figure 3B). Top markers for one of these clusters (*Myh4*, *Pvalb*, *Mybpc2*, *Actn3*, and *Atp2a1*) are typical of type IIb myonuclei, whereas for the other muscle cluster (*Myh7*, *Myl2*, *Myl3*, *Tnnc1*, Tnnt1, *Tnni1*) are characteristic of type I myonuclei^11^^,^^12^ (Figures 3C, 3D, and S3B; Table S4). Therefore, these two muscle clusters could readily be annotated as “Type IIb Muscle” and “Type I Muscle”. These data show that slow and fast muscle type specifications are first detectable in the diaphragm during fetal development. Interestingly, snRNA-seq data from mouse hindlimb muscle reported a small number of nuclei with *Myh4* and *Myh7* expression at E18.5^17^, whereas in diaphragm muscle abundant expression of these genes was detected (Figure 3D). This suggests early maturation of diaphragm during development, consistent with the immediate need of functional respiration right after birth. Because spots composing these clusters were scattered in random patterns throughout the diaphragm muscle (Figure 3B), we conclude that this specification does not display horizontally discernable regionalization, consistent with the scattered distribution of fast and slow fibers in mature murine diaphragm.^48^ We further noted that costal diaphragm predominantly contained spots of type IIb muscle cluster, whereas most of the spots located at the crural diaphragm represented type I muscle cluster (Figure 3B). By manually counting the spots in the two samples, we found that the ratio of type I to type IIb was about 2:1 in crural diaphragm and about 1:2 in costal diaphragm (Figure S3D). This indicates that the two muscles composing the diaphragm exhibit distinct muscle fiber specifications, which might reflect the functional divergence between these two diaphragm domains.^49^ In contrast, the third muscle cluster was specifically located in the ventral part of the costal diaphragm (“Ventral Diaphragm”) (Figure 3B). This region preferentially expressed embryonic muscle markers *Myh3* and *Myog*, the AChR gamma subunit (*Chrng*), early myofibrillogenesis genes (*Nrap*, *Enah*, and *Flnc*) and another less mature peripheral muscle marker at E14.5 *Csrp3* (Figures 3A and S3B; Table S4). This suggests that the ventral diaphragm was less developed at day E18.5 compared to the other regions. Taken together, these spatial expression profiles not only indicate different fiber type compositions in crural and costal diaphragm muscles, but also point toward a differential dorsoventral developmental progression of fiber differentiation in costal diaphragm muscle.

#### Comparison of E18.5 gene expression to E14.5

To further explore developmentally regulated gene expression in diaphragm muscle, we integrated E14.5 and E18.5 datasets (Figure S4A) and analyzed GO enrichment in biological processes terms with the top 200 DEGs (based on avg_log2FC) between muscle clusters of E14.5 and E18.5 diaphragms. These data revealed terms related to RNA processing and splicing (GO: 0008380, GO: 0006397), chromatin remodeling (GO:0006338), DNA replication (GO: 0006260), and nuclear division (GO: 0000280) for E14.5, versus ATP metabolism (GO:0046034, GO: 0006754), oxidative phosphorylation (GO: 0006119), electron transport chain (GO: 0022900), and cellular respiration (GO:0045333) for E18.5 (Figure S4B; Tables S5 and S6). Cell cycle gene scores decreased, while muscle energy metabolism transcripts *Ckm* and *Ckmt2* increased from E14.5 and E18.5 (Figure S4C). This was accompanied by a decline in expression of genes involved in myonuclear positioning (*Kif5b*^50^ and *Macf1*^51^) and three tubulin beta genes involved in cell cycle^52^ (*Tubb6*, *Tubb2b*, *Tubb2a*) from E14.5 to E18.5 (Figure S4D), suggesting a decrease in myonuclear movements and/or cytoskeletal organization during development. Conversely, expression of muscle type specifying myosin genes (*Myh7*, *Myh4*, *Myh2*, *Myh1*, *Myl2*, *Myl3*) markedly increased between E14.5 and E18.5. While fast muscle troponin transcripts *Tnni2*, *Tnnt3*, and *Tnnc2* increased, slow muscle troponin *Tnnc1*, *Tnnt1*, and *Tnni1* expression declined or did not change (Figure S4D). Overall, these data reflect the switch from a proliferative state in embryonic diaphragm at E14.5 to a functionally differentiated state in fetal diaphragm at E18.5.

### Identification of genes spatially correlated with AChR expression

**Figure 4:**
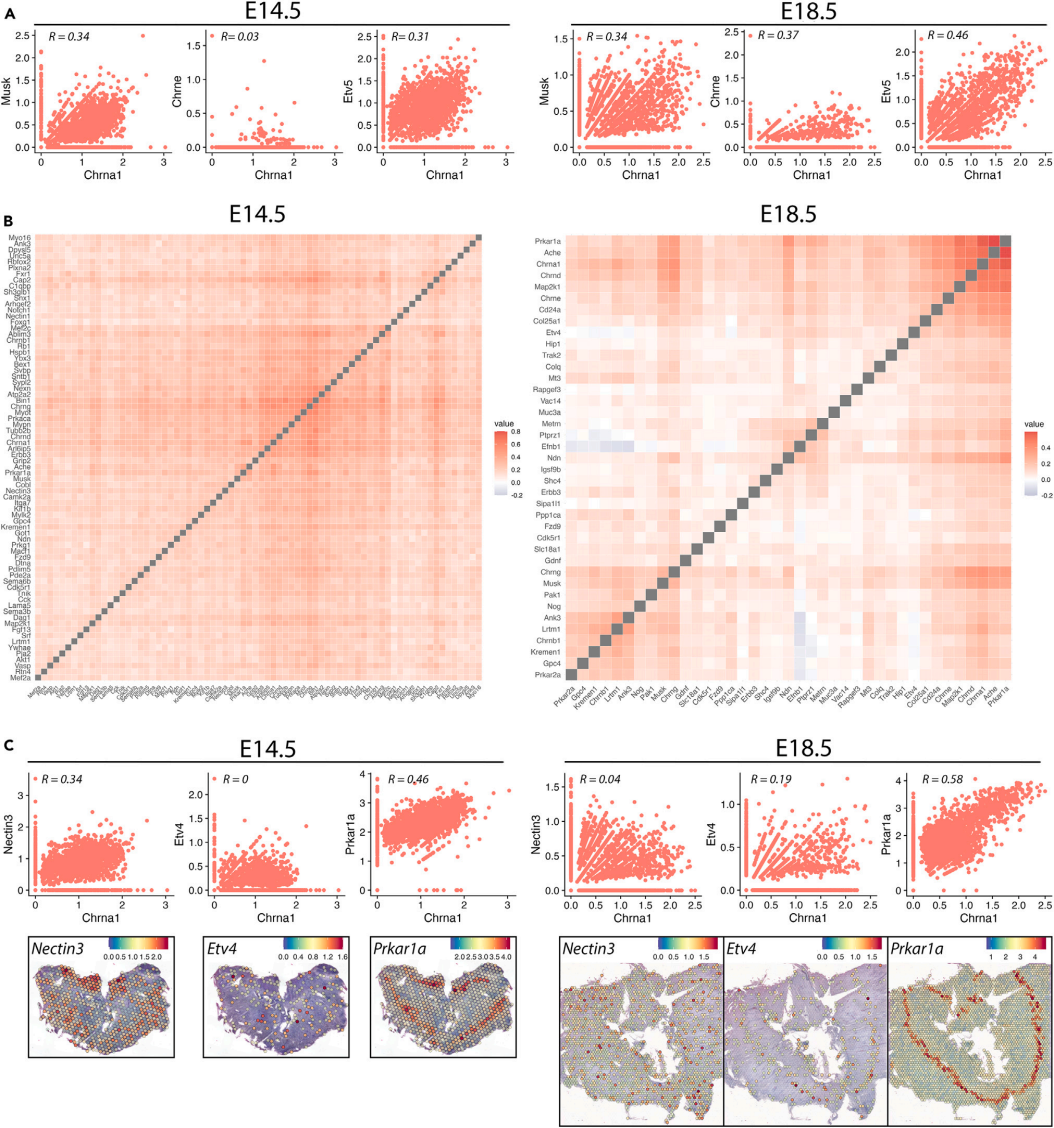
Correlation of synapse and axon genes with *Chrna1* (A) FeatureScatter plots demonstrating correlation between *Musk*, *Chrne* and *Etv5* with *Chrna1* expression levels in the E14.5 (left) and E18.5 (right) spatial dataset; each dot represents one capture spot, and the axes show the expression values of indicated genes. (B) Correlation matrix (Pearson) of genes associated with synapse development and neuron projection within the top 500 highest correlations with *Chrna1* at E14.5 (left) and at E18.5 (right). Diagonally ordered gray squares represent correlation value of 1 for the same genes. (C) FeatureScatter plots demonstrating correlation between *Nectin3*, *Etv4* and *Prkar1a* with *Chrna1* in the E14.5 (left) and E18.5 (right) spatial dataset and SpatialFeaturePlots of these genes.

Expression of many genes involved in NMJ formation is characteristic for the muscle center and identification of such molecules enabled researchers to describe essential molecular mechanisms of the development of the NMJ and of synapse formation at large.^2^^,^^3^^,^^4^ For example, *Etv5*^22^ and *MuSK*^21^ mRNAs are restricted to the muscle center and their expression in this domain is crucial for proper gene expression in subsynaptic nuclei and thus to establish neuromuscular synaptic patterning. Mouse embryonic diaphragm has been a prime model for the study of NMJ formation due to its flat morphology and stereotypical motor innervation pattern. Thus, having spatial transcriptomics data from embryonic mouse diaphragms prompted us to identify novel genes showing similar expression patterns as AChRs during the period of NMJ patterning. To this end, we determined the Pearson correlation of all covered genes with *Chrna1* transcript at E14.5 and E18.5 diaphragms (Table S7). FeatureScatter plotting of known NMJ genes with *Chrna1* indicated that in such datasets moderate correlation can be considered as biologically significant. For example, correlation values for *Chrna1* and *Musk*, a critical determinant for central AChR patterning, was around 0.34 at both E14.5 and E18.5 (Figure 4A) (Table S7). Due to the wide expression of *Chrna1* throughout the E14.5 diaphragm, the number of highly correlated genes was higher compared to the clearly separated domains at E18.5 (Figure S4E; Table S7), resulting from less Visium spots co-expressing *Chrna1* and other genes. Among the top 500 genes highly correlated with *Chrna1* expression, we focused our attention on the genes associated with GO terms neuron projection development and synapse (retrieved from Jax informatics at https://www.informatics.jax.org/index.shtml) because such genes expressed by muscle are expected to play potential roles in building postsynaptic structures, guiding motor axons to establish central innervation pattern and retrogradely trigger presynaptic differentiation of the motor axon terminals. Examples of such *Chrna1*-correlated genes are: At E14.5: *Cap2*, *Prkaca*, *Ablim3*, *Nectin3*, *Itga7*, *Macf1*, *Vasp*, *Dag1*, *Sema6b*, at E18.5: *Efnb1*, *Cd24a*, *Mt3*, *Etv4*, *Metrn*, *Trak2*, *Vac14*, *Shc4*, *Sipa1l1:* and at both developmental stages: *Prkar1a*, *Map2k1*, *Lrtm1*, *Ndn*, Ank3 (Figure 4B). In Figure 4C, *Nectin3* represents an example for centrally expressed genes only at E14.5, *Etv4* only at E18.5 and *Prkar1a* at both developmental stages. In addition, these data revealed correlations of centrally expressed genes with each other, which might be suggestive of their regulation by shared upstream pathways and/or their functions in common processes. For example, at E18.5 *Ank3*, *Lrtm1*, *Chrnb1*, *Kremen1*, *Gpc4*, *Prkar2a* show high correlation with each other; *Prkar1a*, *Ache*, *Chrna1*, *Chrnd*, *Map2k1* represent another such group of correlated genes (Figure 4B, right). This novel dataset is indicative of the highly complex molecular regulation of nerve-muscle synapse formation, and it represents a valuable resource for developing testable hypotheses regarding the potential involvement of each of these molecules in neuromuscular synaptogenesis. Additionally, we considered that genes negatively correlated with *Chrna1* might also represent physiologically important genes in that exclusion of axon guidance molecules specifically from the central NMJ region might indicate their roles in determining the central innervation pattern. However, by visually inspecting the top 500 negatively correlated genes, we failed to detect even a single gene with such an expression pattern.

### Compromised diaphragm muscle development in Ca_V_1.1^−/−^ mice

**Figure 5:**
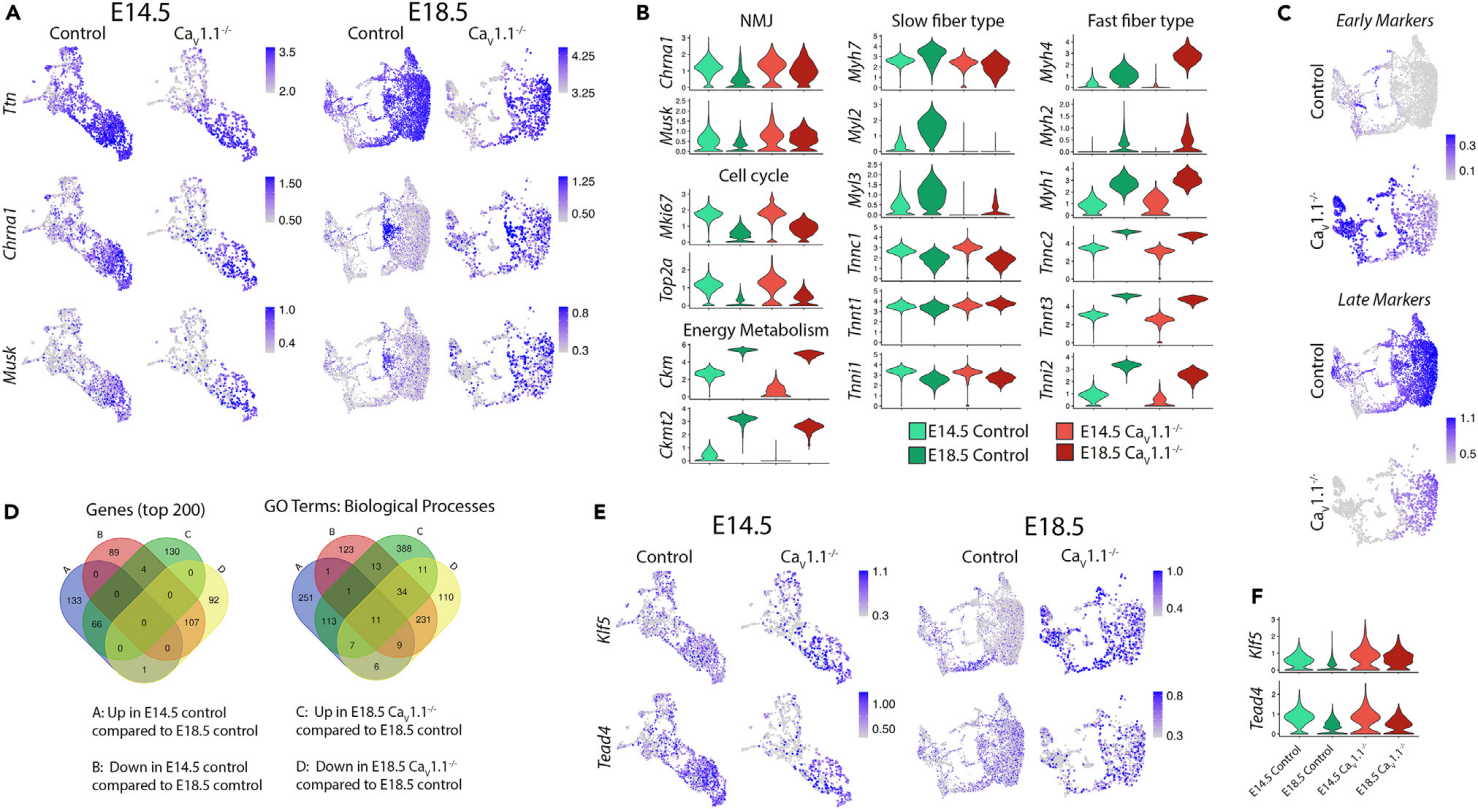
Spatial transcriptomics reveals aberrant regulation of myogenic genes in Ca_V_1.1^−/−^ mice (A) FeaturePlots showing expression of *Ttn* (muscle), *Chrna1* and *Musk* (NMJ) displayed by UMAP in control and Ca_V_1.1^−/−^ integrated dataset at E14.5 (left) and E18.5 (right). (B) Violin plots showing expression of representative genes differentially expressed in control and Ca_V_1.1^−/−^ samples at E14.5 and E18.5. Y axis indicates expression level. (C) FeaturePlots of module scores of muscle differentiation markers displayed by UMAP in E18.5 control and Ca_V_1.1^−/−^ integrated dataset show increased expression of early markers (top) and a decreased expression of late markers (bottom) in Ca_V_1.1^−/−^ muscles. (D) Venn diagrams of top 200 DEGs genes and GO terms for these genes between E14.5 control and E18.5 control and between E18.5 Ca_V_1.1^−/−^ and E18.5 control muscles indicate more shared genes and GO terms for upregulated genes in E18.5 Ca_V_1.1^−/−^ with E14.5 control muscle and for downregulated genes in E18.5 Ca_V_1.1^−/−^ with E18.5 control muscle. (E) FeaturePlots showing expression of *Klf5* and *Tead4* displayed by UMAP in control and Ca_V_1.1^−/−^ integrated dataset at E14.5 (left) and E18.5 (right). (F) Violin plots of *Klf5* and *Tead4* expression in muscle clusters of E14.5 and E18.5 control and Ca_V_1.1^−/−^ spatial datasets. Y axis indicates expression level.

The skeletal muscle L-type calcium channel Ca_V_1.1, also known as skeletal muscle dihydropyridine receptor (DHPR), is the voltage-sensor of excitation-contraction coupling^6^ and one of the key regulators of activity-dependent processes in skeletal muscle, NMJ and tendon development.^8^^,^^53^^,^^54^^,^^55^^,^^56^^,^^57^ Its expression is specific to skeletal muscle.^58^ Despite lacking Ca_V_1.1, skeletal muscles of *dysgenic* (*mdg*/*mdg*)^59^ mice show regular spontaneous electrical activity but lack activity-dependent calcium signals and mechanical contractions.^6^ Therefore, we performed spatial transcriptomics experiments on one diaphragm each of E14.5 and E18.5 mice lacking Ca_V_1.1. We integrated the Ca_V_1.1^−/−^ datasets with the control datasets at corresponding time points (Figure S5A), then performed standard clustering workflow, and assessed the effects of disrupted excitation-contraction coupling on gene expression in developing muscle. Ca_V_1.1 depletion resulted in increased expression of the synaptic markers *Musk* and *Chrna1* (Figures 5A and 5B), which are suppressed by muscle activity in extrasynaptic regions of developing skeletal muscles.^55^^,^^56^^,^^60^ In Ca_V_1.1^−/−^ diaphragm at E18.5 but not E14.5 *Chrna1* and *Musk* expression was no longer restricted to the center of the muscle fibers, but broadly expressed in Ttn-expressing capture spots, which represent skeletal muscle (Figures 5A and S5B).

#### Transcriptional profile of Ca_V_1.1^−/−^ mice recapitulates truncated muscle development

Because Ca_V_1.1^−/−^ mice display aberrant muscle development^59^ and cell cycle exit is important for proper myogenesis,^61^ we analyzed cell cycle scores and expression of cell cycle markers (*Mki67*^62^ and *Top2a*^63^), all of which were found to be enhanced at both E14.5 and E18.5 (Figures 5B and S5C). This was accompanied by upregulation of genes encoding histone proteins in Ca_V_1.1^−/−^ muscle at both E14.5 and E18.5 (Table S8). Furthermore, two of the important energy metabolism genes, *Ckm* and *Ckmt2*, showed a strong reduction in the absence of Ca_V_1.1 (Figure 5B). We hypothesized that genes that mark early or late embryonic muscle development (Table S5) would be deregulated if muscle development is delayed or truncated in Ca_V_1.1^−/−^ mice at later developmental stages. An “Early markers” module score was generated with the top 100 genes (based on avg_log2FC) whose expression declined from E14.5 to E18.5, and a “Late markers” module score was generated with the top 100 genes whose expression increased from E14.5 to E18.5 in controls. As expected, FeaturePlots for these module scores revealed an increase in early markers and a decrease in late markers in Ttn-expressing capture spots of E18.5 Ca_V_1.1^−/−^ diaphragm compared to that of E18.5 controls (Figure 5C). This is also displayed in the heatmaps for these genes (Figure S8A). We performed GO analysis for the top 200 DEGs in E18.5 Ca_V_1.1^−/−^ muscles (Table S9). The biological processes GO terms for upregulated genes matched those for E14.5 compared to E18.5 controls (Figure S4B), mostly related to RNA processing. Conversely, terms for downregulated genes in E18.5 Ca_V_1.1^−/−^ muscle were similar to terms for E18.5 compared to E14.5 (Figure S4B), such as ATP metabolism and cellular respiration. Venn diagrams also highlighted more shared genes and GO terms between upregulated genes in E18.5 Ca_V_1.1^−/−^ muscles and early (E14.5) markers, and between downregulated genes in E18.5 Ca_V_1.1^−/−^ muscles and late (E18.5) markers (Figure 5D). These data are consistent with the notion that muscle differentiation in Ca_V_1.1^−/−^ mice is truncated at the end of embryonic development, because the lack of activity-induced calcium signals and contractile activity fails to downregulate early-stage muscle genes and upregulate late-stage muscle genes during myogenesis. Additionally, consistent with the recent snRNA-seq study of E18.5 Ca_V_1.1^−/−^ hindlimb muscles,^17^ we also observed increased *Klf5* but normal *Tead4* expression in Ca_V_1.1^−/−^ diaphragm (Figures 5E and 5F; Table S8), indicating a shared activity-dependent mechanism involving Klf5 function by hindlimb and diaphragm muscles during muscle development.

#### Impact of Ca_V_1.1 knockout on muscle contractile gene expressions

Ca_V_1.1 knockout also led to notable diverging changes in myosin and troponin gene expressions. Whereas slow myosin heavy and light chain genes (*Myh7*, *Myl2*, *Myl3*) appeared to be strongly downregulated in Ca_V_1.1^−/−^ diaphragm muscle, this was not the case for slow muscle type troponin genes (*Tnnc1*, *Tnnt1*, and *Tnni1*). Fast muscle type troponins (*Tnnc2*, *Tnnt3*, and *Tnni2*) were slightly changed in mutant muscle, whereas fast myosin heavy chains (*Myh4*, *Myh1*, *Myh2)* showed a strong increase in expression at E18.5 Ca_V_1.1^−/−^ muscles (Figure 5B). These data indicate that these muscle fiber type genes are differentially controlled by Ca_V_1.1-dependent calcium signaling and excitation-contraction coupling.

### Effects of Ca_V_1.1 knockout on synaptogenic gene expression

**Figure 6:**
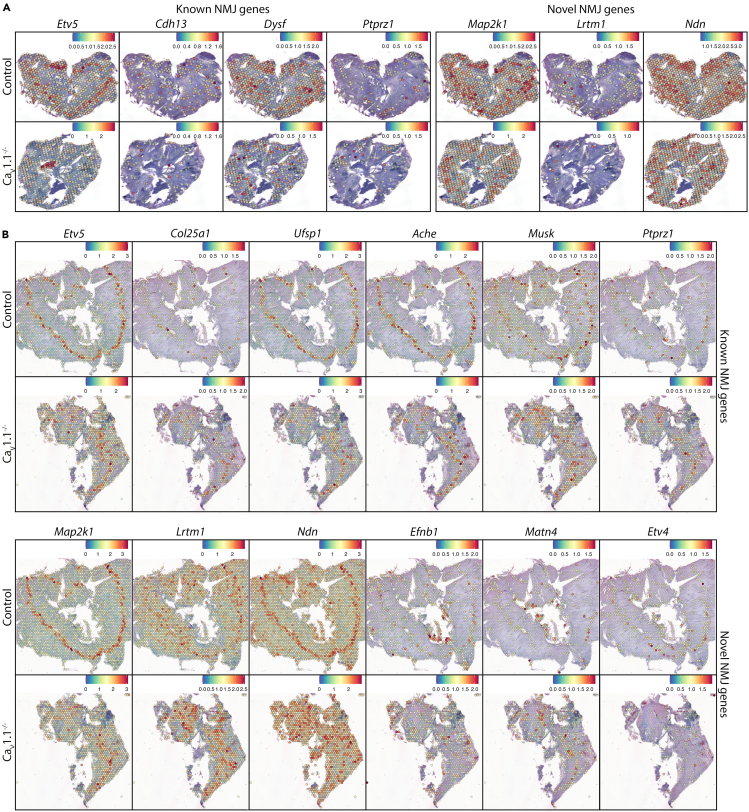
Spatial expression of synapse and axon related genes that show altered distribution by Ca_V_1.1 knockout (A) SpatialFeaturePlots demonstrating expression levels and distributions of affected genes in E14.5 control and E14.5 Ca_V_1.1^−/−^ diaphragm. (B) SpatialFeaturePlots demonstrating expression levels and distributions of affected genes in E18.5 control and E18.5 Ca_V_1.1^−/−^ diaphragm.

Because of the established roles of Ca_V_1.1 in postsynaptic AChR clustering and patterning as well as retrograde regulation of presynaptic differentiation of the motor axons at the NMJ,^54^^,^^55^^,^^56^^,^^57^^,^^64^ we were particularly interested in identifying axon- and synapse-related genes whose expression levels and/or distribution in muscle are affected by Ca_V_1.1 function. Analysis of DEGs between NMJ clusters of control and Ca_V_1.1^−/−^ diaphragms at E18.5 followed by clusterProfiler GO (Table S10) revealed no terms related to synaptic processes and only a small number of terms related to axonogenesis for genes downregulated in Ca_V_1.1^−/−^ mice. On the other hand, a large number of upregulated genes associated with axonogenesis- and synapse-related terms were detected in Ca_V_1.1^−/−^ mice. This is consistent with the increased number, wide distribution, and the precocious maturation of NMJs accompanied by increased motor innervation in the absence of neuromuscular synaptic activity^7^^,^^65^ or Ca_V_1.1 function at E18.5.^55^ Because NMJs in Ca_V_1.1^−/−^ diaphragms become broadly scattered throughout the muscle fibers, also the genes involved in NMJ formation and motor innervation downstream of Ca_V_1.1 are expected to display a dispersed distribution pattern. Therefore, we visually inspected the distribution of synaptic genes revealed by GO analysis and genes classified as centrally expressed (Figure 4) together with *Chrna1* in order to assess their potential regulation by Ca_V_1.1 during NMJ formation. Although the thinner and narrower diaphragm muscles of dysgenic Ca_V_1.1^−/−^ mice and the related difficulty of obtaining complete horizontal sections, made it more challenging to analyze the distribution of the transcripts of interest, a number of genes associated with NMJ formation showed clearly irregular location and altered expression levels in E14.5 and E18.5 mutant muscle. A complete list of such genes with comments is provided in the Table S11; representative examples are shown in Figure 6. The affected genes include a number of crucial players of NMJ development and function, whose distribution and/or level of expression were impacted by Ca_V_1.1 knockout at E14.5 and/or E18.5, such as *Etv5*,^22^ *Col25a1*,^66^ *Cdh13*,^67^ *Ufsp1*,^12^ *Ache*,^23^ *Macf1*,^68^ and *Musk*.^69^ In addition, this analysis also revealed several novel molecules with centrally enriched expression in controls but not Ca_V_1.1 diaphragms, such as *Map2k1*, *Sema6b*, *Lrtm1*, *Ndn*, *Efnb1*, *Matn4*, *Etv4* (Figures 6 and S5D). This differential expression pattern is suggestive of their roles in NMJ formation and their regulation by Ca_V_1.1.

### Compromised muscular and neuro-muscular development in muscle-specific β-catenin knock-out mice

#### Multiple roles of β-catenin in diaphragm muscle development

**Figure 7:**
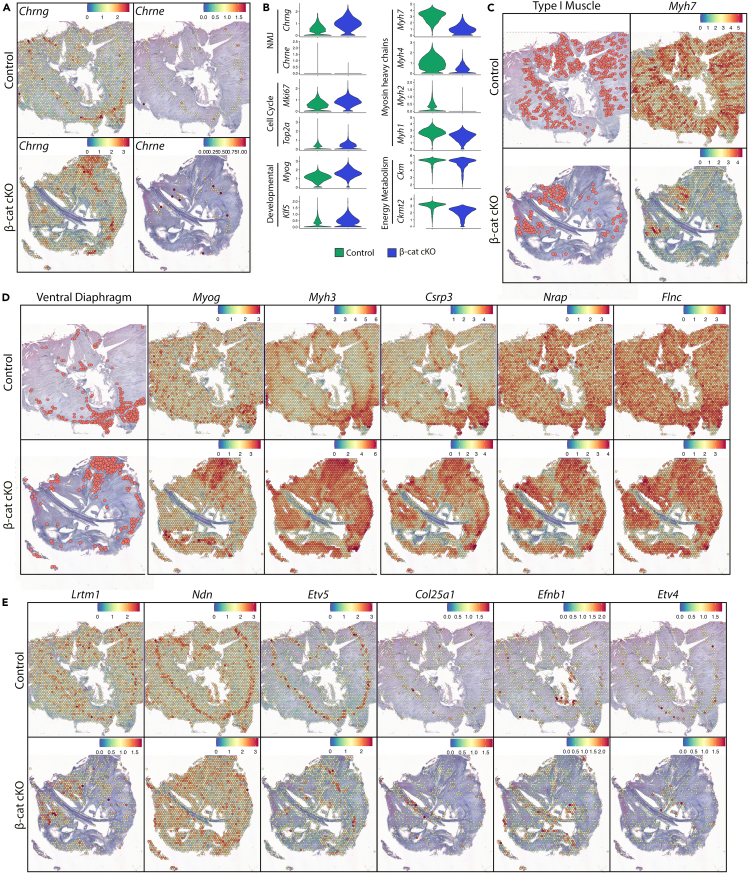
Spatial regulation of gene expression by β-catenin (A) SpatialFeaturePlots demonstrating expression levels and distributions of *Chrng* and *Chrne* in E18.5 control and E18.5 β-cat cKO diaphragms. (B) VlnPlots of expression levels of selected representative genes in clusters annotated as muscle in control and β-cat cKO diaphragm. Y axis indicates expression level. (C) Distribution of “Type I Muscle” cluster (left) and *Myh7* (right) expression in E18.5 control and β-cat cKO diaphragm shows a drastic reduction of slow muscle cluster and *Myh7* expression in costal diaphragm of β-cat cKO samples. (D) Distribution of “Ventral Diaphragm” cluster and expression of associated genes (*Myog*, *Myh3*, *Csrp3*, *Nrap*, *Flnc*) in E18.5 control and β-cat cKO diaphragm show the aberrant localization of the ventral diaphragm cluster and associated genes in the mutant muscle. (E) SpatialFeaturePlots demonstrating expression levels and distributions of synaptogenic genes in E18.5 control and β-cat cKO diaphragm.

We have recently shown that concomitant lack of Ca_V_1.1 and β-catenin at E18.5 leads to severe malformation of diaphragm muscle,^64^ suggesting a direct involvement of β-catenin in the regulation of muscle development. Thus, we performed spatial transcriptomics on E18.5 diaphragm from mice with conditional depletion of β-catenin in muscle tissues, (β-Cat cKO).^64^^,^^70^ We integrated two E18.5 control samples and one E18.5 β-cat cKO sample (Figure S6A), performed standard clustering and applied DEGs analysis in muscle clusters of control and β-Cat cKO samples. Among the top upregulated genes in β-cat cKO sample, *Chrng* captured our attention (0.74401 avg_log2FC, 5E-182 p_val_adj) (Table S12). While this embryonic AChR gamma isoform was highly expressed, the mature AChR epsilon subunit *Chrne* was virtually absent in β-Cat cKO diaphragm (Figures 7A and 7B), indicating a role of β-catenin in AChR subunit switch. Cell cycle scores (Figure S6B) and expression of cell cycle markers (*Mki67* and *Top2a*) (Figure 7B) were higher in β-cat cKO muscle, indicating a more proliferative state. This was accompanied by increased expression of developmental genes *Myog* and *Kfl5*, and decreased expression of mature muscle genes *Myh7*, *Myh4*, *Myh2*, *Myh1*, *Ckm*, and *Ckmt2* (Figure 7B). Early markers were increased, and late markers were decreased in E18.5 β-cat cKO compared to control samples (Figures S6B and S8B). GO analysis for 328 downregulated genes revealed association mostly with energy metabolism (Tables S12 and S13). Type I muscle genes were considerably reduced in costal diaphragm but not in crural diaphragm of β-cat cKO and accompanied by a strong reduction in *Myh7* in costal diaphragm (Figure 7C). This indicates the involvement of β-cat cKO in the specification of the slow myogenic program in costal diaphragm, but not in crural diaphragm. Most of the spots that expressed markers for immature muscle and early myofibrillogenesis, were located in the dorsal part of the costal diaphragm in the mutant (Figure 7D, left column). The characteristic localization of genes marking the ventral diaphragm cluster in controls (such as *Myog*, *Myh3*, *Csrp3*, *Flnc*, *Nrap*) (Figure 7D) and mature muscle markers such as (*Ckm*, *Ckmt2*, *Atp2a1*) (Figure S6C) and corresponding early and late markers (Figure S6D, left) appeared to be disrupted in the β-cat cKO, emphasizing delayed muscle development in these mice. Seurat cell cycle modules did not show regional alterations (Figure S6D, right), indicating that the disrupted dorsoventral maturity pattern in the mutant diaphragm was not due to an increased proliferative state in the absence of muscle β-catenin. Additionally, central accumulation of *Myh3* mRNA was not observed in the mutant (Figure 7D). Altogether, the spatial gene expression profile of the β-cat cKO diaphragm points to a massive dysregulation of muscle development, including the loss of AChR subunit switch, regulation of energy metabolism, fiber type specification and the failure to establish the dorsoventral expression pattern in the mouse diaphragm in the absence of β-catenin.

#### Synaptogenic gene expression controlled by β-catenin

The other process where β-catenin plays a pivotal role in muscle is NMJ formation^70^ and we have shown that β-cat cKO cooperates with Ca_V_1.1 to regulate AChR clustering and motor innervation.^64^ Thus, we explored how knockout of β-catenin affected the expression and/or distribution of genes potentially involved in NMJ formation, by analyzing DEGs between NMJ clusters of control and the mutant diaphragm (Table S14) and performed GO analysis to extract genes involved in axonogenesis and synapse organization, neuron projection development and synapse using clusterProfiler (Jax informatics at https://www.informatics.jax.org/index.shtml). 114 genes were up- and 49 genes were downregulated in the NMJ cluster of β-cat cKO muscle (Figure S7A). Among the genes upregulated in E18.5 β-cat cKO, only *Chrna1* and *Chrng* were expressed in the central muscle domain. On the other hand, many of the genes downregulated (such as *Lrtm1*, *Ndn*, *Etv5*, *Col25a1*, *Mt3*, *Trak2*, *Metrn*, *Map2k1*, *Ank3*, *Ache*) in E18.5 β-cat cKO were expressed in the central region (Figures 7E and S7B). This suggests that β-catenin largely possesses a gene expression promoting activity rather than a suppressing activity on the NMJ-specific transcription in subsynaptic nuclei. Although expression of *Slit2* was proposed to be a muscle-derived signal for motoneuron differentiation downstream of β-catenin expressed in the muscle center of mouse diaphragm,^71^ we failed to detect such a distribution or regulation by β-catenin (Figure S7C). On the other hand, comparison with the spatial transcriptomics data from Ca_V_1.1^−/−^ diaphragm, (Figures 6, 7E, and S7B) revealed hitherto unnoticed molecules that are regulated by both β-catenin and Ca_V_1.1, such as *Lrtm1*, *Ndn*, *Etv5*, *Col25a1*, *Efnb1*, *Etv4*. Since β-catenin and Ca_V_1.1 cooperate in multiple ways in regulating distinct features of NMJ formation,^64^ the data revealing the impact of β-catenin and Ca_V_1.1 on expression levels and patterns of these centrally expressed genes provide valuable leads for studying how NMJ formation is regulated by these two key regulators of muscle development.

## Discussion

This study reports the first spatially resolved transcriptomic data on embryonic (E14.5) and fetal (E18.5) mouse diaphragm muscle, as a primary model system for studying the mechanisms of neuro-muscular junction formation. We retrieved horizontally discernable domains of muscle, NMJ, MTJ and tendons, all of which were resolved in respective spatial gene expression patterns. Based on differential expression patterns, the muscle tissue could be further sub-divided into costal and crural muscles, as well as in radially (along the longitudinal axes of the muscle fibers) and dorsoventral regions of costal diaphragm, revealing functionally specialized domains and a spatial progression of muscle differentiation. Comparison of the spatial gene expression patterns with embryonic and/or fetal diaphragm muscles of transgenic mouse models with a global depletion of Ca_V_1.1^−/−^ and a muscular depletion of β-catenin, as key regulators of muscle development and NMJ formation, recapitulated known deficits in muscle development and NMJ formation in mutant mouse diaphragm and revealed new differentially expressed candidate genes possibly involved in the spatiotemporal regulation of these processes.

In recent studies, global gene expression profiles emerged from single nucleus RNA and ATAC sequencing in embryonic, fetal and adult muscle and spatial transcriptomics in adult limb muscle.^10^^,^^11^^,^^12^^,^^13^^,^^14^^,^^17^^,^^72^ While these studies provide highly valuable information on the regulation of myogenesis and muscle differentiation by sequentially active transcription factors and programs, they cannot provide information on the spatial progression of these processes, both laterally in different muscle regions and longitudinally along the length of the myofibers. Our datasets assessing murine developing diaphragm muscles enabled us to characterize spatial transcriptomic differences between the embryonic and fetal developmental stages. While immature muscle regions, characterized by the canonical marker *Myh3*, were preferentially located in the peripheral regions of the muscle (i.e., the ends of the muscle fibers) at E14.5, clusters with corresponding expression profiles and associated genes, such as *Csrp3*, *Flnc*, and *Nrap*, were restricted to ventral diaphragm at E18.5. Second, separation of muscle clusters into clusters with specific type I and IIb fiber type expression profiles is only observed at the fetal stage. Third, some synapse formation- and neuron projection-related genes display high spatial correlation with the nicotinic acetylcholine receptor gene *Chrna1* only at E14.5 and others at E18.5. Such dynamic changes of gene expression patterns between E14.5 and E18.5 show that the complex regulation of muscle differentiation and its role in the formation of NMJ with motor nerves needs to consider both, developmental and spatial regulation.

The interplay of different signaling pathways is illustrated by the effects of Ca_V_1.1 or β-catenin knockouts on gene expression for myogenesis.^64^ Our current data demonstrate that at late embryonic/fetal development, both mutants fail to show the down- and upregulation of genes associated with muscle differentiation in control diaphragm. The central function of Ca_V_1.1 in activity-dependent muscle differentiation is well-known and our spatial transcriptomics analysis recapitulated some of the striking features, like the failed downregulation of synapse-specific genes in extra-synaptic muscle regions. Also, the role of β-catenin in muscle development has been suggested previously and that the two regulatory pathways clearly cooperate with one another. Although initial characterization of β-cat cKO mice reported normal gross muscle morphology,^70^ overexpression of β-catenin leads to formation of ectopic muscle tissues in central tendon of the diaphragm,^73^ indicating the direct involvement of β-catenin in the myogenesis of the diaphragm. Moreover, the concomitant knockout of Ca_V_1.1 or β-catenin results in severe muscle developmental defects at E18.5.^64^ Our current data suggest that multiple and unexpected aspects of myogenesis are controlled by β-catenin. Localization of the developmentally less matured clusters, classified as ventral diaphragm in controls, is disrupted in β-cat cKO mice, suggesting a role of β-catenin in establishing the dorsoventral patterning of the diaphragm. Wnt/β-catenin signaling is well-known to be involved in positional tissue patterning^74^ and our data indicate a novel regulation of such process in muscles by β-catenin. Moreover, the absence of β-catenin leads to substantial reduction in slow type I fiber cluster. Surprisingly, this reduction is seen only in costal diaphragm, while crural diaphragm is protected from it. Why these two muscle domains are affected differentially by β-catenin knockout warrants further investigation. The finding that *Lrtm1* expression is strongly reduced in the costal but not in the crural domain of β-cat cKO diaphragm (Figure 7E), makes it a strong candidate for a downstream effector of β-catenin signaling for slow muscle development. Indeed, a recent study reported a role of *Lrtm1* in muscle development.^75^ Additionally, it has been elegantly documented that Pax7-driven deletion of β-catenin affects secondary myotube formation in the limb.^76^ Consistent with this, central enrichment of *Myh3* expression, an indication of secondary myotube formation associated with motor innervation, was not detected in β-cat cKO diaphragm. This observation strongly suggests that β-catenin contributes to the initiation of the secondary myotube formation at the central muscle domain of the diaphragm. However, this and other observed defects in β-cat cKO diaphragm, such as AChR subunit switch, might be an indirect consequence of reduced motor innervation in the mutant diaphragm^64^^,^^70^ and can be resolved in future studies. Altogether, the spatial transcriptional profile of β-cat cKO diaphragm provides novel perspectives for future studies on regulation of diaphragm muscle development by β-catenin.

During embryonic development, NMJs are formed by spatio-temporally regulated molecular mechanisms. Prior to nerve arrival, muscle-intrinsic mechanisms concentrate AChRs and MuSK at the centrally located prospective synaptic regions, to which motor axons are guided. Arrival of the nerve provides signals to regulate the innervation-dependent fine-tuning of this NMJ patterning. At the same time, muscle-derived retrograde signals control axon pruning and presynaptic differentiation, such as the concentration of synaptic vesicles at the active zones, in register with postsynaptic structure.^4^ However, it is not known to what extent molecular mechanisms are shared by these consecutive steps of NMJ development. For example, prior to innervation LRP4/MuSK signaling determines the pre-patterning of AChRs and other NMJ proteins in the center of the muscle fibers. After innervation this signaling mechanism is enhanced by nerve-derived agrin secreted at the NMJ, whereas spontaneous and nerve-induced muscle activity and calcium signaling keep AChR and MuSK expression spatially in check.^77^^,^^78^ Conversely, a multiplicity of retrograde signals from muscle to nerve are thought to control the guidance of the motor axon to the synaptic zones, the accumulation of the presynaptic molecules and structures at the nerve terminals, as well as synapse elimination. In order to identify these regulatory mechanisms, it is important to explore the expression of which molecules are spatio-temporally regulated in muscle during early and late stages of NMJ formation. Our spatial transcriptomics datasets provide such information comprehensively by reporting gene expression that are spatially correlated with *Chrna1* at E14.5 and E18.5. Notably, some molecules are indeed spatially co-expressed with AChRs at both time points, suggestive of their functions during initial and later steps of the NMJ formation. On the other hand, other molecules showed clear differences in their early and late expression patterns. For example, *Etv4*, which belongs to the Pea3 transcription factor family and plays a role in retrograde signaling during axonal growth,^79^ is centrally expressed specifically at E18.5, suggesting its function as a transcriptional factor in subsynaptic nuclei during later steps of NMJ formation.

Datasets from Ca_V_1.1^−/−^ and β-cat cKO diaphragms represent additional sources of information for a better understanding of NMJ development since these molecules play diverse roles during NMJ formation. We have recently shown that lack of either Ca_V_1.1 or β-catenin results in smaller AChR clusters distributed over a wider region of muscle fibers at E14.5, whereas at E18.5 AChR clusters are larger in Ca_V_1.1^−/−^ diaphragms and normal in β-cat cKO diaphragms. On the other hand, the roles of Ca_V_1.1 and β-catenin in postsynaptic muscle counteract each other in the retrograde regulation of presynaptic motor nerve branching.^64^ Therefore, molecules whose expressions are impacted by both Ca_V_1.1 and β-catenin knockout, might represent strong candidates as regulators of NMJ formation downstream of Ca_V_1.1 and β-catenin. For example, *Col25a1* expression in muscle is regulated by activity and required for motor innervation.^66^ Here, we show that *Col25a1* is overexpressed, and its distribution is disrupted in Ca_V_1.1^−/−^ diaphragm, but *Col25a1* is downregulated in β-cat cKO diaphragm. Therefore, molecular mechanisms for motor innervation may include a counteractive regulation of *Col25a1* expression by Ca_V_1.1 and β-catenin, in order to determine both, sufficient innervation and its correct pattern. Such opposing transcriptional regulation by Ca_V_1.1 and β-catenin, also demonstrate the specificity of the two signaling pathways in regulating muscle differentiation as opposed to their common action of delaying development. Dysregulated expression of *Etv5* might also contribute to the defects observed in both Ca_V_1.1^−/−^ and β-cat cKO diaphragms. Our data demonstrate a regulation of *Etv5*, which has been shown to be an important transcriptional regulator of NMJ genes including AChR subunits and MuSK.^22^ Moreover, T-cadherin has been reported to have an inhibitory effect on motor axon growth,^67^ and we have observed a strong downregulation of T-cadherin (*Cdh13*) expression in Ca_V_1.1^−/−^ diaphragms at E14.5. Reduction of such an inhibitory cell adhesion molecule in Ca_V_1.1^−/−^ diaphragms might also contribute to the unrestricted motor nerve branching and innervation observed in *dysgenic* mice. It is important to note that activity-dependent regulation of AChR clustering and motor innervation is largely mediated by Ca_V_1.1. Mice lacking Ca_V_1.1 function or Ca_V_1.1-driven calcium signaling mimic most of the defects observed in mice where neuromuscular synaptic or muscle activity is blocked.^7^^,^^55^^,^^56^^,^^57^^,^^65^ Yet lack of Ca_V_1.1 function leads to even increased muscle’s electrical activity.^55^ Calcium is the only secondary messenger that is able to translate electrical activity into cellular response and L-type calcium channels are well-known for their roles in coupling excitation with transcription. Thus, our datasets from Ca_V_1.1^−/−^ mice are more generally applicable to activity-dependent transcriptional regulation during myogenesis and neuromuscular synaptogenesis.

In conclusion, our spatial transcriptomics datasets deliver a powerful resource of the first spatially resolved global gene expression in the embryonic and fetal muscle in the mouse embryo. Moreover, it presents evidence for regional changes of gene expression patterns in the developing diaphragm (center vs. periphery, costal vs. crural, dorsal vs. ventral). Together with data from mice lacking critical regulators of myogenesis and neuromuscular synaptogenesis (Ca_V_1.1 and β-catenin), this report provides valuable insights and numerous testable hypothesis for the future investigations on muscle and NMJ development.

### Limitation of the study

Current technology does not allow spatial transcriptomics analysis at single cell resolution. Therefore, the clusters identified in this study captured transcripts from multiple cell types, which also resulted in several undefined clusters in the small E14.5 diaphragm. Nonetheless, this does not prevent us to spatially resolve the main anatomical structures and domains of the diaphragm. Moreover, it must be noted that the small size and fragility of tissue from embryonic mice and even more so from mutants affecting development limit the use of this approach. Because obtaining horizontal sections of the mutant diaphragm was challenging, some important data points are lacking or only documented with a single sample. Nevertheless, using three E14.5 and two E18.5 control samples, we consistently observed the same expression patterns, making us confident that also the conclusions drawn from the data points with limited samples are meaningful and will provide testable hypotheses for future functional studies. Finally, the analysis in this study is limited to muscle tissue since it represents our prime interest. However, our publicly available datasets can be readily used for analyses of other reported tissue types and the effects of Ca_V_1.1 and β-catenin knockouts thereon. For example, Ca_V_1.1 function has been shown to be involved in tendon development and impacts of Ca_V_1.1 knockout on gene expression in tendons, or tools for ligand-receptor interaction analysis such as CellChat,^80^ can provide insight in muscle contraction-dependent tendon development.

## STAR★Methods

### Key resources table

**Table:** 

REAGENT or RESOURCE	SOURCE	IDENTIFIER
**Critical commercial assays**		

Visium Spatial for FFPE Gene Expression Kit, Mouse Transcriptome	10X Genomics	1000339
Visium Mouse Transcriptome Probe Kit - Small	10X Genomics	1000365
Visium FFPE Reagent Kit - Small	10X Genomics	1000361
Visium Spatial Gene Expression Slide Kit	10X Genomics	1000188

**Deposited data**		

Raw and Processed Data	This paper	GSE244014

**Experimental models: Organisms/strains**		

Ca_V_1.1^−/−^ Mice	N/A	Pai, 1965^59^; Tanabe et al. 1988^6^
HSA-Cre; β-catenin^flox/flox^	N/A	Li et al. 2008^70^; Kaplan & Flucher 2022^64^

**Software and algorithms**		

SpaceRanger (2.0.1)	10X Genomics	https://www.10xgenomics.com/support/software/space-ranger/latest
R (4.2.2)	Open Source	https://www.r-project.org
Seurat (4.3.0)	Hao et al. 2021^31^	https://satijalab.org/seurat/articles/spatial_vignette
clusterProfiler (4.6.0)	Wu et. 2021^45^	https://guangchuangyu.github.io/software/clusterProfiler/
Clustree (0.5.0)	Zappia & Oshlack, 2018^32^	https://github.com/lazappi/clustree
Biorender	Biorender	https://www.biorender.com

**Other**		

Analysis Code	This paper	https://github.com/kaplanmm/diaphragm_spatial/blob/main/embryonic%20diaphragm%20spatial.R

### Resource availability

#### Lead contact

Further information and requests for resources and reagents should be directed to and will be fulfilled by the lead contact, Mehmet Mahsum Kaplan (mehmet.kaplan@iem.cas.cz).

#### Materials availability

This study did not generate new unique reagents.

#### Data and code availability

• Raw and processes data and Seurat objects loaded by Load10X_Spatial function for all the samples have been deposited in GEO and will be publicly available at the time of publication with accession number GSE244014.
• This paper does not produce original codes. The codes used for data analysis can be accessed at https://github.com/kaplanmm/diaphragm_spatial/tree/main.
• Any additional information required to reanalyze the data reported in this paper is available from lead contact upon request.

### Experimental model and study participant details

#### Mice

All animal protocols conformed to the guidelines of the European Community (86/609/EEC) and were approved by the Austrian Ministry of Science (GZ: 2020–0.073.957 and GZ: 2020–0.073.961). Ca_V_1.1 KO and β-cat cKO mice were previously described.^6^^,^^64^^,^^70^ Ca_V_1.1 KO mice were obtained by heterozygous crossing of Ca_V_1.1^+/−^ mice or by HSA–β-cat^floxed/+^; Ca_V_1.1^+/−^ mice and β-cat^floxed/floxed^; Ca_V_1.1^+/−^. β-cat KO mice were obtained by crossing HSA–β-cat^floxed/+^; Ca_V_1.1^+/−^ mice. β-cat^floxed/floxed^; Ca_V_1.1^+/−^. Control mice contained at least one Ca_v_1.1 and one β-catenin expressing allele. Sperm plugs were checked daily at 8:00 a.m. and the day sperm plug was detected was counted as E0.5. Embryos were harvested at E14.5 or E18.5 as indicated in the figures.

### Method details

#### Spatial transcriptomics and library preparation

Trunks of the harvested embryos were stored in 4% PFA prepared in phosphate buffer (pH: 7.2) until further use, which did not exceed 4 weeks. Diaphragms were dissected in PBS and placed in biopsy bags maintaining its flat shape for dehydration. Next day, diaphragms were flat-embedded in paraffin and stored at 4°C until sectioning. 5 μm thick *en face* sections of FFPE diaphragms were checked for tissue quality (completeness, section plane) and collected for RNA analysis on Visium FFPE slides. Tissue sections were processed for H&E staining and imaged with a Zeiss Imager Z2 microscope (Zeiss, Vienna, Austria) equipped with a Pixelink PL-D674CU-CYL-07451 camera and processed using TissueFAXS software version 7.137 (Tissue Gnostics, Vienna, Austria). Libraries were generated from cDNA following the manufacturer’s instructions and checked with both a Qubit 2.0 Fluorometer (Invitrogen, Carlsbad, CA) and an Agilent Bioanalyzer DNA assay (Agilent Technologies, Santa Clara, CA). Subsequently, libraries were sequenced in paired-end 150bp mode on a NovaSeq 6000 system (Illumina, San Diego, CA).

### Quantification and statistical analysis

Visium Spatial Transcriptomics sequencing data were aligned using the default SpaceRanger (2.0.1) pipeline for FFPE slides in a Singularity Container running Ubuntu 22.04 on a high-performance cluster (Medical University of Innsbruck). All data were aligned against the mouse reference genome (MM10) provided by 10x Genomics and the Visum_Mouse_Transcriptome_Probe_Set_v1.0_mm10-2020-A.csv as background. Visium Slide Images were reoriented according to their position and slide spot locations and annotations downloaded from 10xGenomics.

Outputs from SpaceRanger were analyzed with Seurat (4.3.0) first loaded into R (4.2.2) environment by Load10X_Spatial function. This was followed by SCTransform normalization and data integration with IntegrateData function. We used 30 principal components for dimensionality reduction and for both tSNE and UMAP as non-linear dimensional reduction embedding. FindNeighbours and FindClusters methods were performed for clustering with resolution set to 0.3, 0.4 or 0,6 for different datasets determined by inspecting Clustree (0.5.0) graph. SCT assay was used for spatial plot visualization of the features. Normalized and Scaled data of the Spatial assay were used for finding markers for clusters and DEGs and generating heatmaps with default settings. Default Wilcoxon Rank-Sum test was used for DEGs. Seurat FindMarkers function was used to extract DEGs between clusters and conditions using default parameters where *p*-values were adjusted based on Bonferroni correction as default for the function. Gene ontology was performed with clusterProfiler R package (4.6.0) and enrichgo function to retrieve gene enrichments for Biological Processes Ontology space with default parameters. Cutoff was set to below 0.05 to adjust for multiple comparison. Clusters were annotated by using previously known markers from experimental data or recent single nucleus RNA sequencing studies cited throughout the manuscript, gene ontology for identified markers and mouse cell atlas for neonatal muscles (https://bis.zju.edu.cn/MCA/search2.html). This paper did not produce original codes, however R codes for an overview of data analysis are provided in https://github.com/kaplanmm/diaphragm_spatial/tree/main. Venn diagrams were generated using online platform at https://bioinformatics.psb.ugent.be/webtools/Venn/.

## Supplemental information

Document S1. Figures S1–S8

Table S1. Quality control metrics of analyzed samples, related to Figures 1, 3, 5, 7

Table S2. E14.5 Seurat Cluster markers, related to Figure 1 List of markers of all identified clusters for three integrated E14.5 diaphragms extracted by FindAllMarkers Seurat Function with default parameters.

Table S3. clusterProfler gene ontology for DEGs between central vs. peripheral clusters, related to Figure 2 ClusterProfiler GO in biological processes run by using DEGs between central muscle and peripheral muscle clusters of three integrated E14.5 diaphragms. DEGs were extracted by FindMarker function where ident.1 and ident.2 arguments were set to central and peripheral clusters, respectively. GO terms were extracted by enrichGO function with default parameters.

Table S4. E18.5 Seurat Cluster markers, related to Figure 3 List of markers of all identified clusters for two integrated E18.5 diaphragms extracted by FindAllMarkers Seurat function with default parameters.

Table S5. DEGs between E14.5 and E18.5, related to Figure 3 DEGs between muscle clusters of three E14.5 and two E18.5 integrated samples, extracted by FindMarker Seurat function where ident.1 and ident.2 arguments were set to E14.5 and E18.5, respectively

Table S6. clusterProfiler gene ontology for DEGs E14.5 vs. E18.5, related to Figure 3 ClusterProfiler GO in biological processes run by using top 200 DEGs between E14.5 and E18.5 (genes retrieved from Table S5). GO terms were extracted by enrichGO function with default parameters.

Table S7. Correlation of covered genes with *Chrna1*, related to Figure 4 Pearson Correlation of all covered genes with *Chrna1* at E14.5 (three integrated samples) and E18.5 (two integrated samples). Spatial assay after normalization and data scaling were used for this analysis.

Table S8. DEGs for muscle clusters of control and Ca_V_1.1^−/−^ samples at E14.5 and E18.5, related to Figure 5 DEGs between muscle clusters of control and Ca_V_1.1^−/−^ samples at E14.5 and E18.5, extracted by FindMarker Seurat function. Positive avg_log2FC indicates downregulation whereas negative avg_log2FC indicates upregulation in Ca_V_1.1^−/−^ muscle compared to controls.

Table S9. clusterProfiler gene ontology for control vs. Ca_V_1.1^−/−^ samples at E18.5, related to Figure 5 ClusterProfiler GO in biological processes run by using top 200 DEGs between muscle clusters of E18.5 control and Ca_V_1.1^−/−^ diaphragms. GO terms were extracted by enrichGO function with default parameters.

Table S10. clusterProfiler gene ontology for NMJ cluster of control and Ca_V_1.1^−/−^ diaphragms, related to Figure 6 clusterProfiler GO in biological processes run by using DEGs between NMJ clusters of E18.5 control and Ca_V_1.1^−/−^ diaphragms. GO terms were extracted by enrichGO function with default parameters. Only synapse and axon related terms are noted.

Table S11. Dysregulated synapse and axon related genes in Ca_V_1.1^−/−^ diaphragm, related to Figure 6 Comments on dysregulated (either aberrant distribution or expression level for E14.5 and E18.5) genes in Ca_V_1.1^−/−^ samples.

Table S12. DEGs for muscle clusters of control and β-cat cKO samples, related to Figure 7 DEGs between muscle clusters of control and β-cat cKO samples at E18.5, extracted by FindMarker Seurat function. Positive avg_log2FC indicates downregulation whereas negative avg_log2FC indicates upregulation in β-cat cKO compared to controls.

Table S13. clusterProfiler gene ontology for genes downregulated in β-cat cKO diaphragm, related to Figure 7 ClusterProfiler GO in biological processes run by using 328 downregulated genes (genes retrieved from Table S12) in muscle clusters of E18.5 control and β-cat cKO diaphragms. GO terms were extracted by enrichGO function with default parameters.

Table S14. DEGs between NMJ cluster of control and β-cat cKO diaphragm, related to Figure 7 DEGs between NMJ clusters of control and β-cat cKO samples at E18.5, extracted by FindMarker Seurat function. Positive avg_log2FC indicates downregulation whereas negative avg_log2FC indicates upregulation in β-Cat cKO compared to controls.
