# Supplementary material for: Spatial transcriptomics in embryonic mouse diaphragm muscle reveals regional gradients and subdomains of developmental gene expression
Source: iScience. 2024 May 17;27(6):110018. doi: 10.1016/j.isci.2024.110018 (PMC11177202; doi:10.1016/j.isci.2024.110018)
Supplement: Document S1. Figures S1–S8 [file mmc1.pdf]

iScience, Volume *27*

Supplemental information

Spatial transcriptomics in embryonic mouse diaphragm muscle reveals regional gradients and subdomains of developmental gene expression

Mehmet Mahsum Kaplan, Maximilian Zeidler, Annabella Knapp, Martina Hölzl, Michaela Kress, Helga Fritsch, Anne Krogsdam, and Bernhard E. Flucher

Figure S1

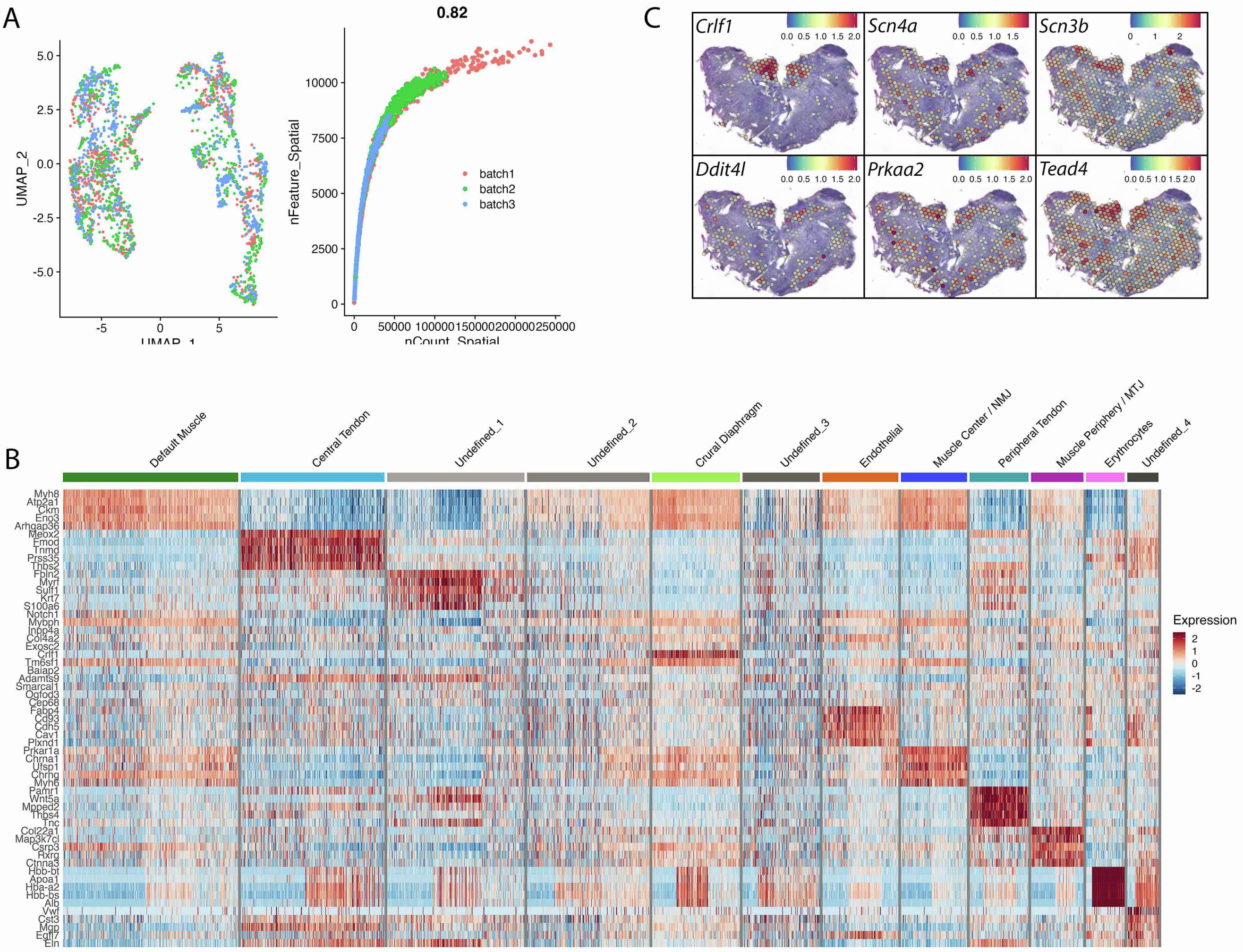

Figure S1: Spatial Transcriptomics reveals gene expression in distinct anatomical regions in E14.5 diaphragms (related to Figure 1)

(A) UMAP representation of three E14.5 control diaphragm samples after batch correction of the integrated data (left) and ScatterFeature plot (right) demonstrating the correlation between the number of genes (nFeature_Spatial) and the number of mRNAs (nCount_Spatial).

(B) Heatmap of top marker genes for each cluster. Each row represents genes labeled on the left; each column represents an individual Visium spot.

(C) SpatialFeaturePlots demonstrating expression levels and distributions of indicated genes.

Figure S2

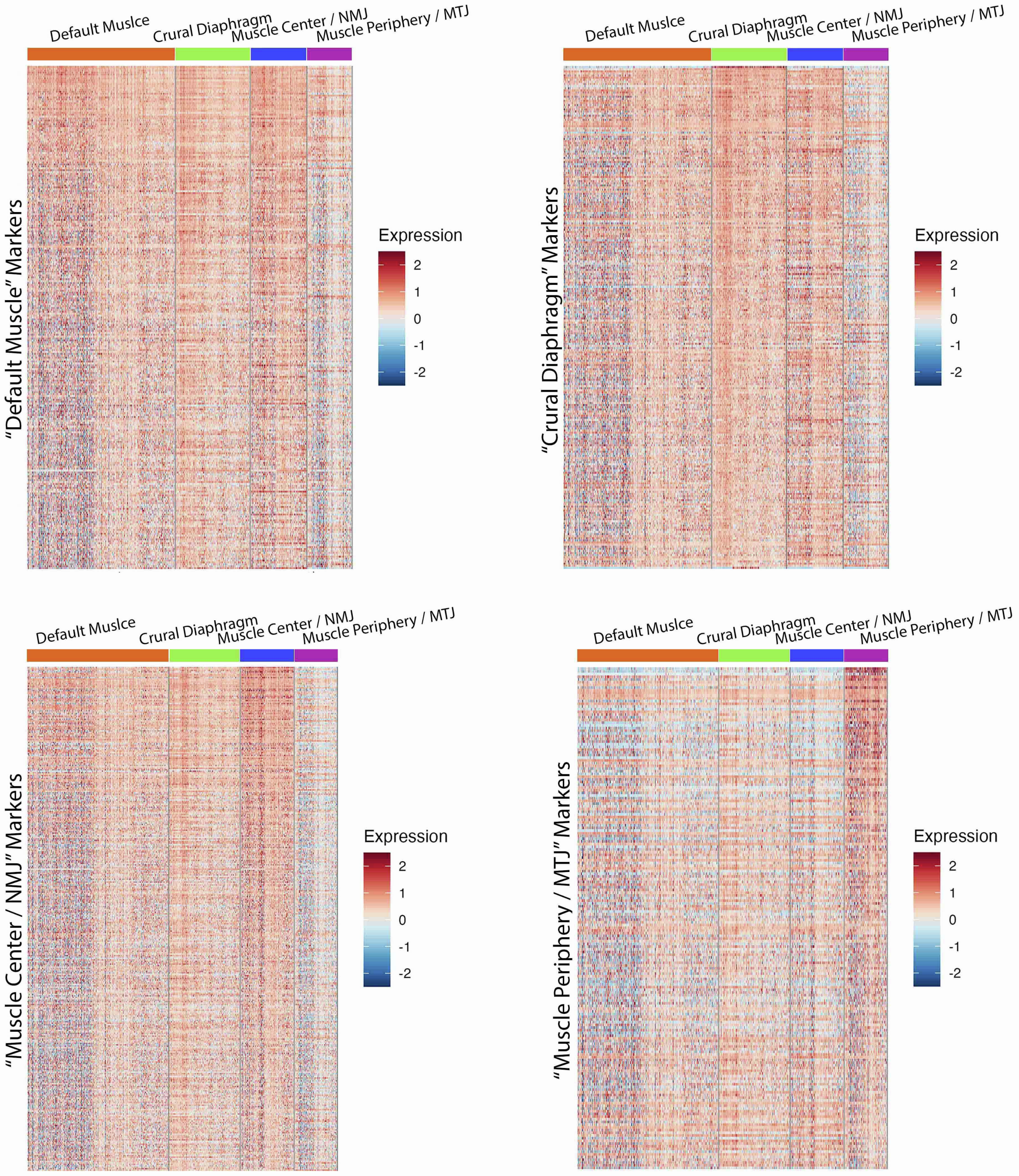

Figure S2: Specialized gene signatures for center and periphery of E14.5 diaphragms (related to Figure 1) Heatmaps of expression levels of markers for "Default Muscle" (top left), "Crural Diaphragms" (top right), "Muscle Center / NMJ" (bottom left) and "Muscle Periphery / MTJ" (bottom right) clusters in all muscle clusters demonstrate unspecialized gene expression signature for default muscle markers, but specialized gene expression profiles in muscle center and muscle periphery clusters.

Figure S3

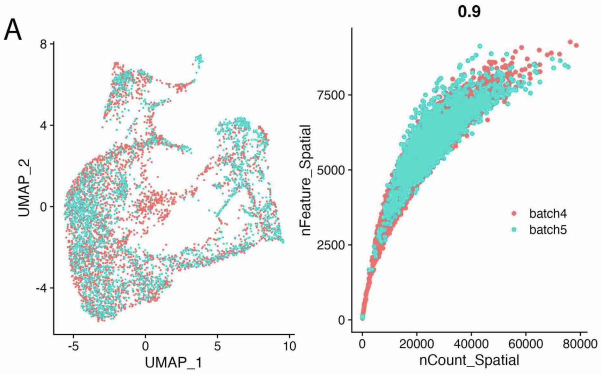

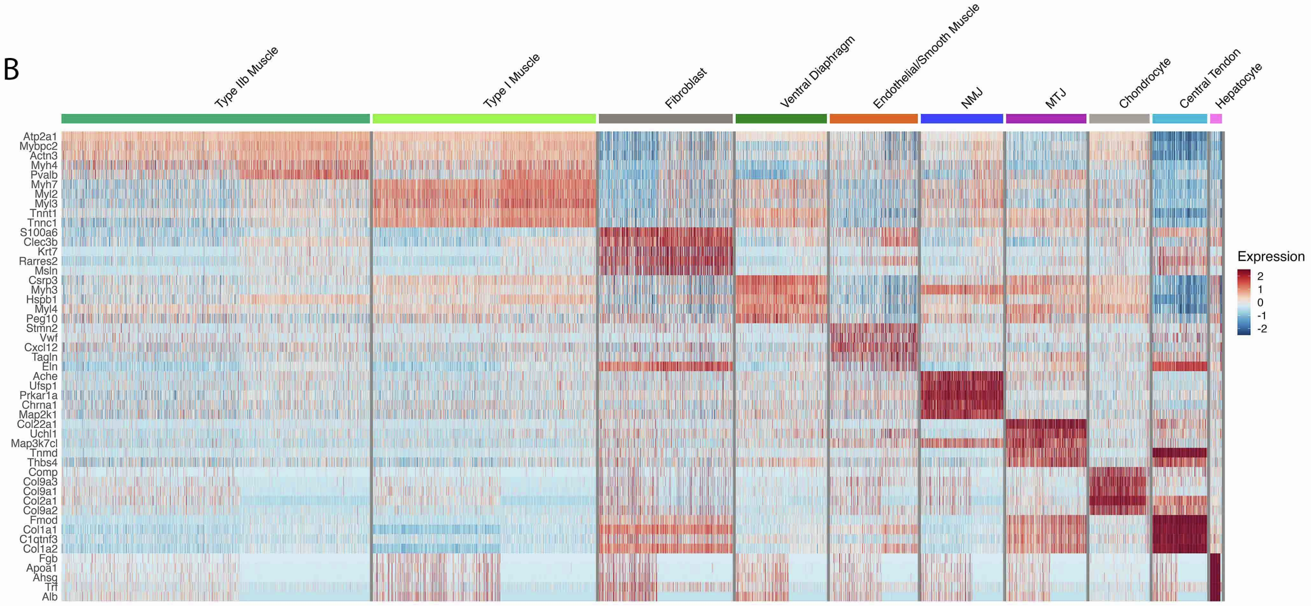

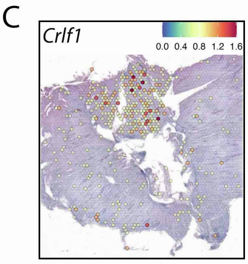

D

	Crural		Costal	
	Type I	Type IIb	Type I	Type IIb
Sample1	212	116	465	817
Sample2	193	161	312	520
	# of spots			

Figure S3: Spatial Transcriptomics of E18.5 diaphragms (related to Figure 3)
(A) UMAP representation of two E18.5 control diaphragm samples after batch correction of the integrated data (left) and ScatterFeature plot (right) demonstrating correlation between number of genes (nFeature_Spatial) and number of mRNAs (nCount_Spatial).
(B) Heatmap of top marker genes for each cluster shows their clear separation from each other. Each row represents genes labeled on the left; each column represents individual Visium spots belonging to the indicated clusters.
(C) SpatialFeaturePlots of expression levels and distributions of Crlf1 at E18.5 showing its specific expression in crural diaphragm portion.
(D) Manual quantification of spots representing slow Type I and fast Type IIb muscle clusters in crural and costal diaphragm domains for two E18.5 control samples.

Figure S4

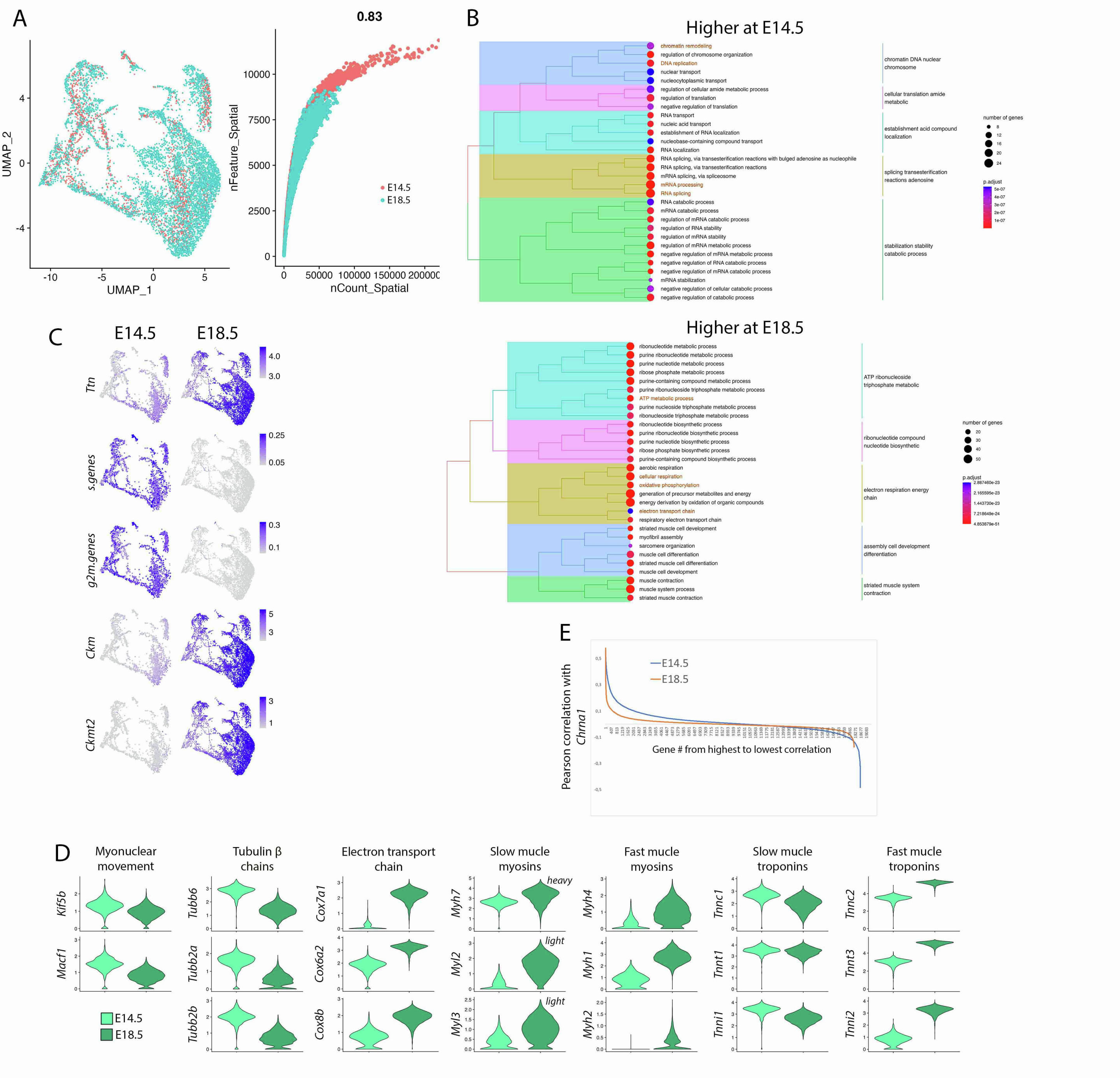

Figure S4: Differential gene expression between E14.5 and E18.5 diaphragms (related to Figures 3 and 4)
(A) UMAP representation of three E14.5 and two E18.5 control diaphragm samples after batch correction of the integrated data (left) and ScatterFeature plot (right) demonstrating the correlation between the number of genes (nFeature_Spatial) and the number of mRNAs (nCount_Spatial).
(B) Tree plots showing the hierarchical clustering of enriched GO terms for the top 200 DEGs with higher expression in E14.5 compared to E18.5 (top) and in E18.5 compared to E14.5 (bottom) diaphragm muscle.
(C) FeaturePlots of Ttn (muscle marker), module scores of cell cycle genes (s.genes for the genes associated with S-phase and g2m.genes for the genes associated with G2M-phase), and energy metabolism genes Ckm and Ckmt2 displayed by UMAP showing a decrease in cell cycle scores and an increase in energy metabolism genes between E14.5 and E18.5.
(D) Violin plots comparing expression of selected differentially expressed genes at E14.5 and E18.5. Y-axis indicates expression levels.
(E) Plotting the Pearson correlation values of all covered genes with Chrna1 indicates higher absolute Pearson correlation values with Chrna1 at E14.5 than at E18.5.

Figure S5

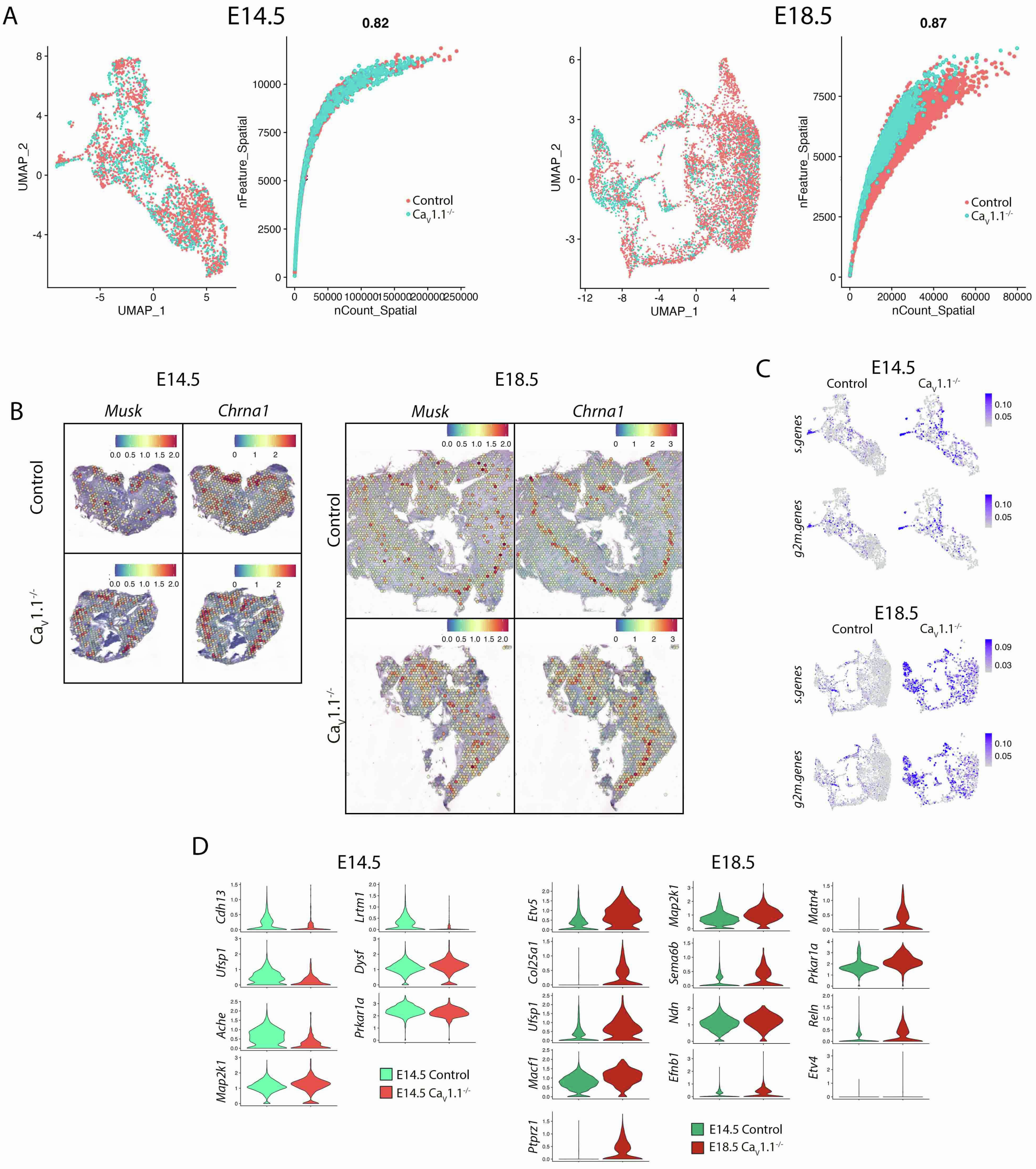

Figure S5: Differences in gene expression between control and $Ca_V1.1^{-/-}$ diaphragm (related to Figure 5)
(A) UMAP representation of three E14.5 control diaphragms and one E14.5 $Ca_V1.1^{-/-}$ diaphragm (left) and two E18.5 control diaphragms and one E18.5 $Ca_V1.1^{-/-}$ diaphragm (right) after batch correction of the integrated data and ScatterFeature plot demonstrating the correlation between the numbers of genes (nFeature_Spatial) and the numbers of mRNAs (nCount_Spatial).
(B) SpatialFeaturePlots demonstrating expression levels and distributions of Chrna1 and Musk at E14.5 (left) and E18.5 (right) control and $Ca_V1.1^{-/-}$ diaphragm.
(C) FeaturePlots of module scores of cell cycle genes (s.genes for the genes associated with S-phase and g2m.genes for the genes associated with G2M-phase) displayed by UMAP in control and $Ca_V1.1^{-/-}$ integrated dataset at E14.5 (top) and E18.5 (bottom) show an increase in cell cycle scores in $Ca_V1.1^{-/-}$ muscles.
(D) Violin plots showing expression of synapse related genes which display differential expression between control and $Ca_V1.1^{-/-}$ samples at E14.5 (left) and E18.5 (right). Y-axis indicates expression level.

Figure S6

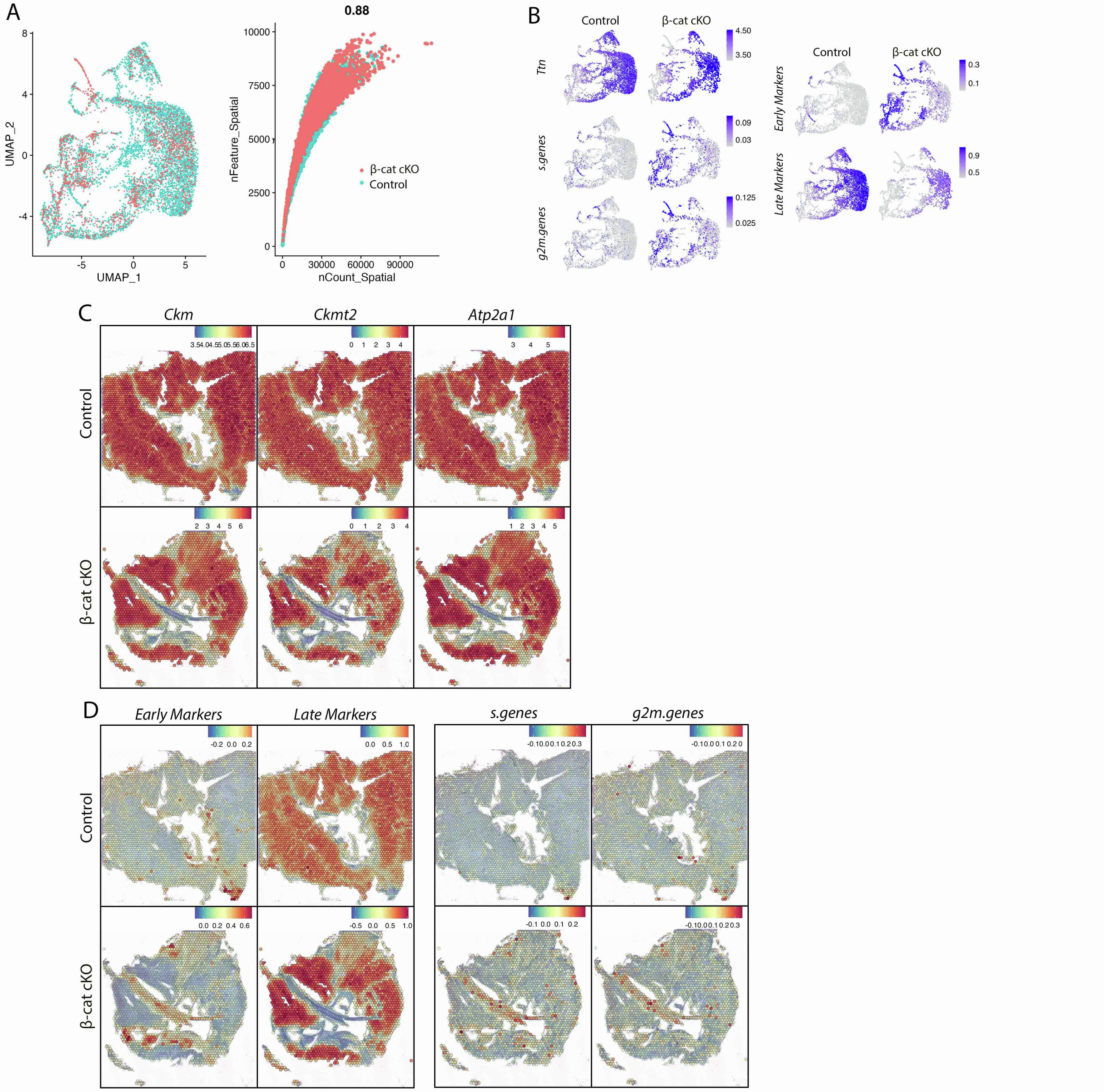

Figure S6: Differential gene expression between control and β-cat cKO diaphragm (related to Figure 7)
(A) UMAP representation of two E18.5 control diaphragms and one E18.5 β-cat cKO diaphragm after batch correction of the integrated data (left) and ScatterFeature plot (right) demonstrating the correlation between the numbers of genes (nFeature_Spatial) and the numbers of mRNAs (nCount_Spatial).
(B) FeaturePlots of Ttn (muscle marker), module scores of cell cycle genes (s.genes for the genes associated with S-phase and g2m.genes for the genes associated with G2M-phase) and early and late markers displayed by UMAP show an increase in cell cycle and early markers scores in E18.5 β-cat cKO muscles, whereas late markers scores decrease.
(C) Expression of mature muscle associated genes (Ckm, Ckmt2, Atp2a1) in E18.5 control and β-cat cKO diaphragm shows the aberrant localization of these genes in the mutant muscle.
(D) Spatial distribution of the early and late module scores (left) and module scores of cell cycle genes (s.genes for the genes associated with S-phase and g2m.genes for the genes associated with G2M-phase) (right) in control and β-cat cKO diaphragms

Figure S7

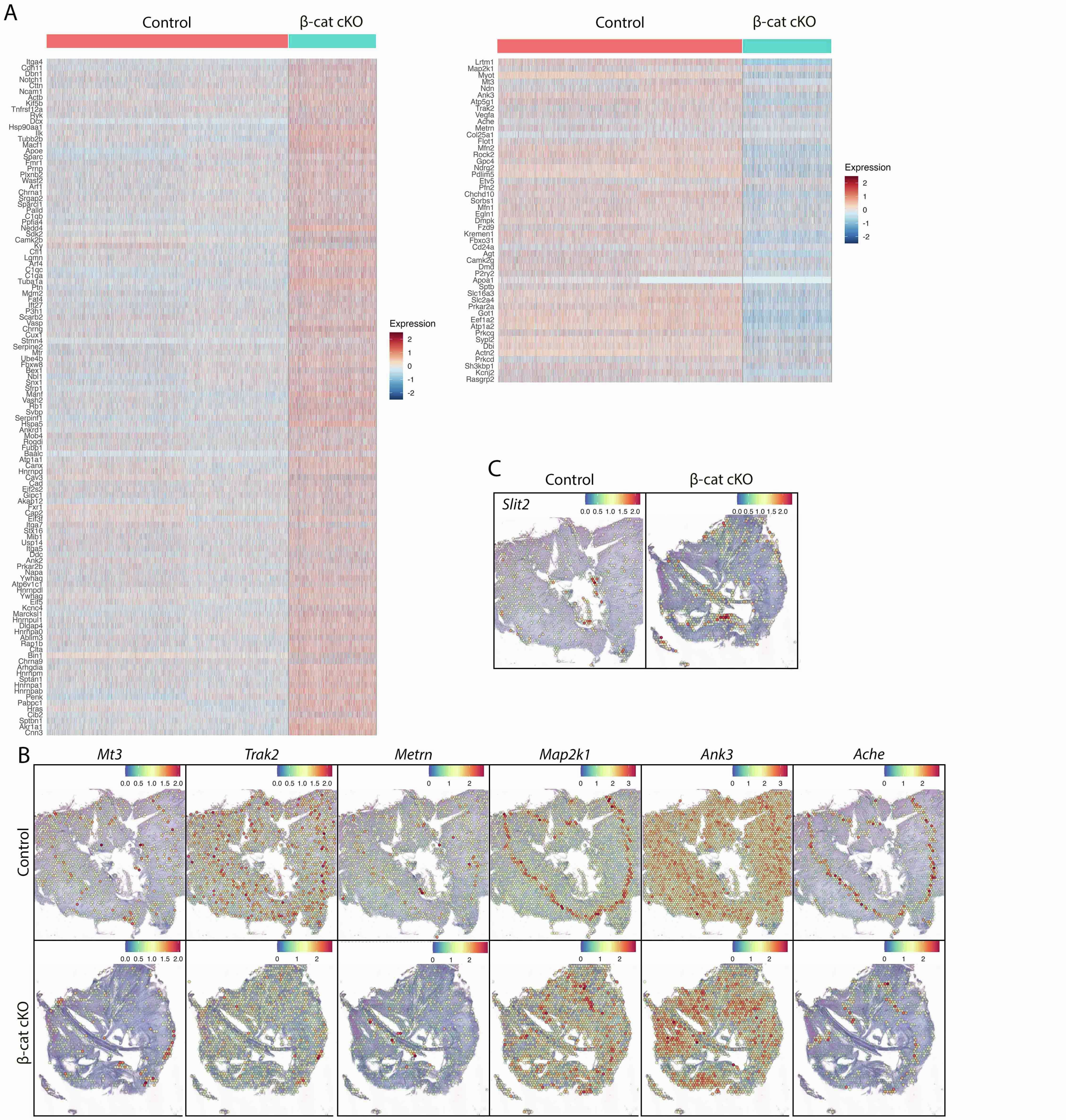

Figure S7: Aberrant expression of synapse and axon related genes in β-cat cKO diaphragm (related to Figure 7)

(A) Heatmap demonstrating expression of upregulated (left) and downregulated (right) synapse and axon related genes in β-cat cKO muscle.

(B) SpatialFeaturePlots showing expression levels and distributions of differentially expressed/distributed NMJ-related genes in E18.5 control and β-cat cKO samples.

(C) SpatialFeaturePlots showing expression levels and distributions of Slit2 in E18.5 control and β-cat cKO muscle.

Figure S8

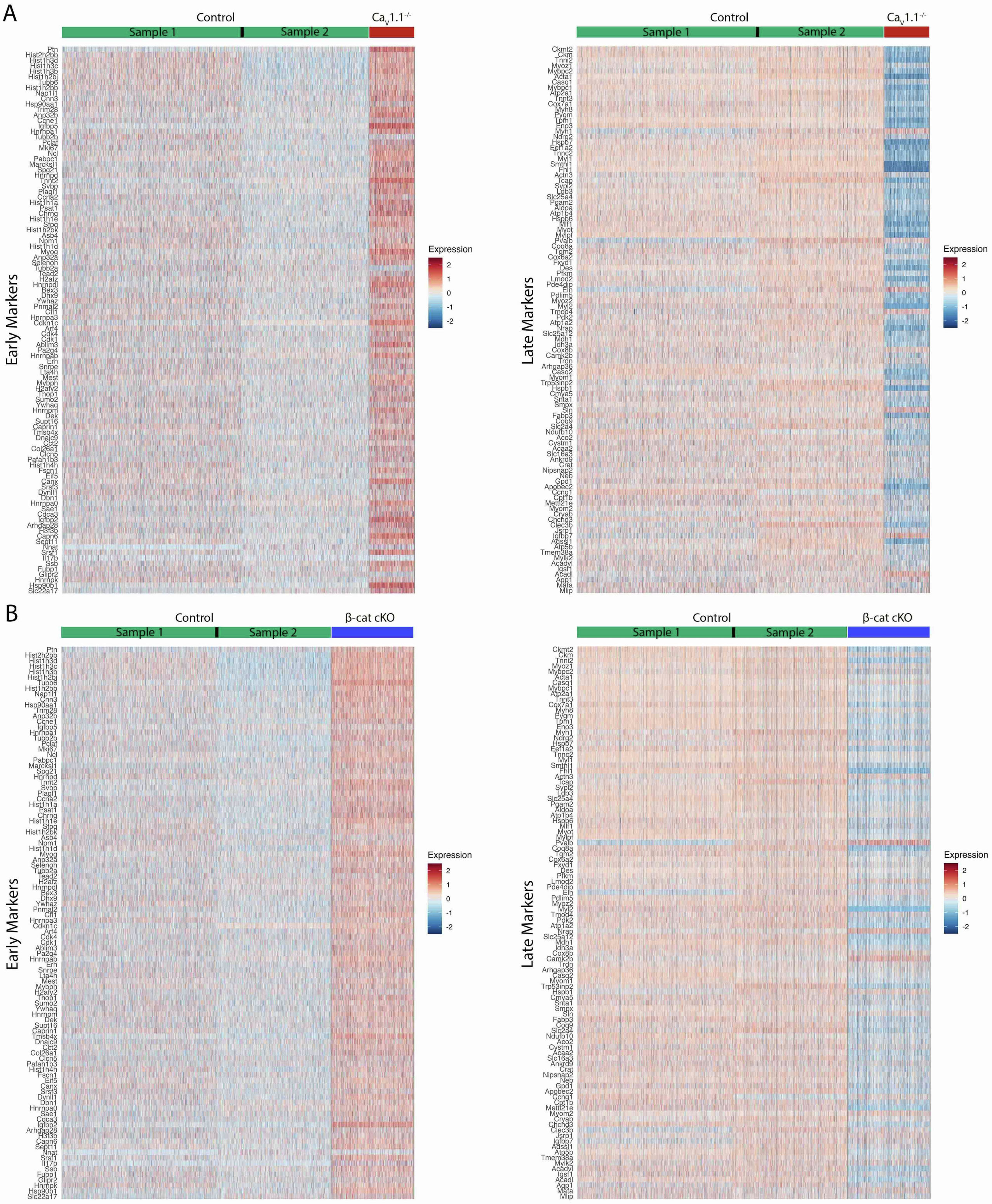

Figure S8: Expression of early and late markers in E18.5 Ca$_V$1.1$^{-/-}$ and β-cat cKO diaphragm (Related to Figure 5 and Figure 7)

(A) Heatmap demonstrating expression of the top 100 early (left) and late (right) markers in E18.5 control and Ca$_V$1.1$^{-/-}$ samples.

(B) Heatmap demonstrating expression of the top 100 early (left) and late (right) markers in E18.5 control and β-cat cKO samples.
